# Supplementary material for: Exploring Mannosylpurines as Copper Chelators and Cholinesterase Inhibitors with Potential for Alzheimer’s Disease
Source: Pharmaceuticals (Basel). 2022 Dec 30;16(1):54. doi: 10.3390/ph16010054 (PMC9864808; doi:10.3390/ph16010054)
Supplement: Supplementary file 1 [file pharmaceuticals-16-00054-s001.zip › pharmaceuticals-2110957-supplementary.pdf]

Commented [M11]: Please carefully check the accuracy of names and affiliations.

Commented [M12]: These author names are different from the Susy, please confirm.

Commented [M13]: If the information provided presents more than one address, please separate the addresses into different affiliations.

Supplementary Materials

Exploring mannosylpurines as copper chelators and cholinesterase inhibitors with potential to Alzheimer's disease

Ignazio Schino [1,2,3], Mariangela Cantore [1,2,3], Modesto de Candia [3], Cosimo D. Altomare [3], Catarina Maria [1,4], João Barros [1], Vasco Cachatra [1], Patrícia Calado [1], Karina Shimizu [5], Adilson A. Freitas [5], Maria C. Oliveira [5], Maria J. Ferreira [5], José N. C. Lopes [5], Nicola A. Colabufo [3]* and Amélia P. Rauter [1,*]

[1] Centro de Química Estrutural, Institute of Molecular Sciences, Faculdade de Ciências, Universidade de Lisboa, Ed C8, Piso 5, Campo Grande, 1749-016 Lisboa, Portugal
[2] Biofordrug srl, Via Dante 95, 70019 Triggiano, Italy
[3] Department of Pharmacy-Pharmaceutical Sciences, University of Bari Aldo Moro, Via E. Orabona 4, 70125 Bari, Italy
[4] Departamento de Química, Faculdade de Ciências e Tecnologia, Universidade Nova de Lisboa, Largo da Torre, 2825-149 Caparica, Portugal
[5] Centro de Química Estrutural, Institute of Molecular Sciences Instituto Superior Técnico, Universidade de Lisboa, Av. Rovisco Pais, 1049-001 Lisboa, Portugal
* Correspondence: aprauter@fc.ul.pt (A.P.R.); nicolaantonio.colabufo@uniba.it (N.A.C.); Tel.: +351-964-408-824 (A.P.R.)

^{1}H NMR and ^{13}C NMR spectra of the new compounds 5-8, 9,10, 12, 13, 15 and 16

Compound **5** (in CD_3OD)

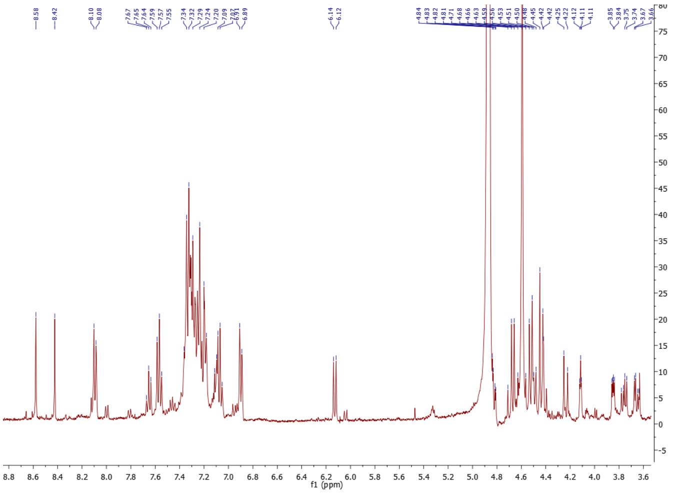

Figure S1. ^{1}H NMR spectrum for compound **5**.

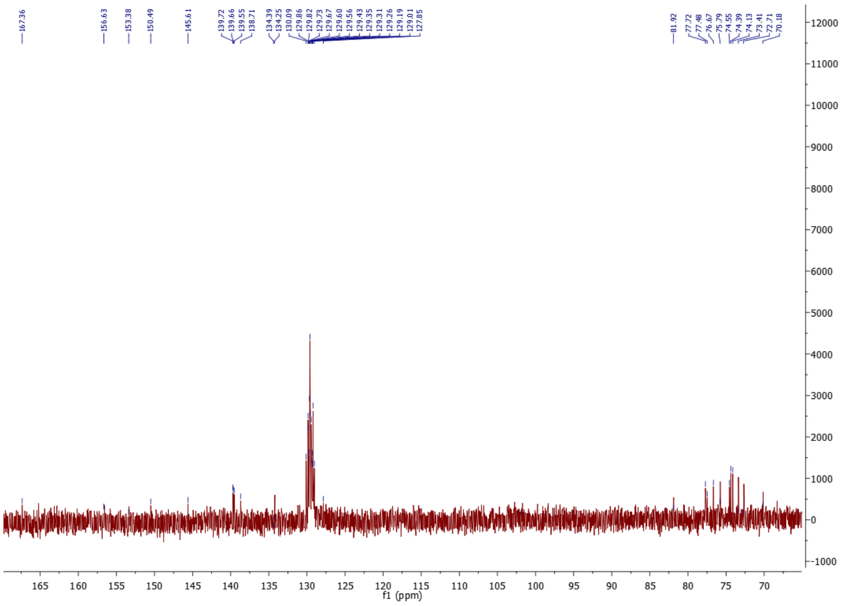

Figure S2. ^{13}C NMR spectrum for compound **5**.

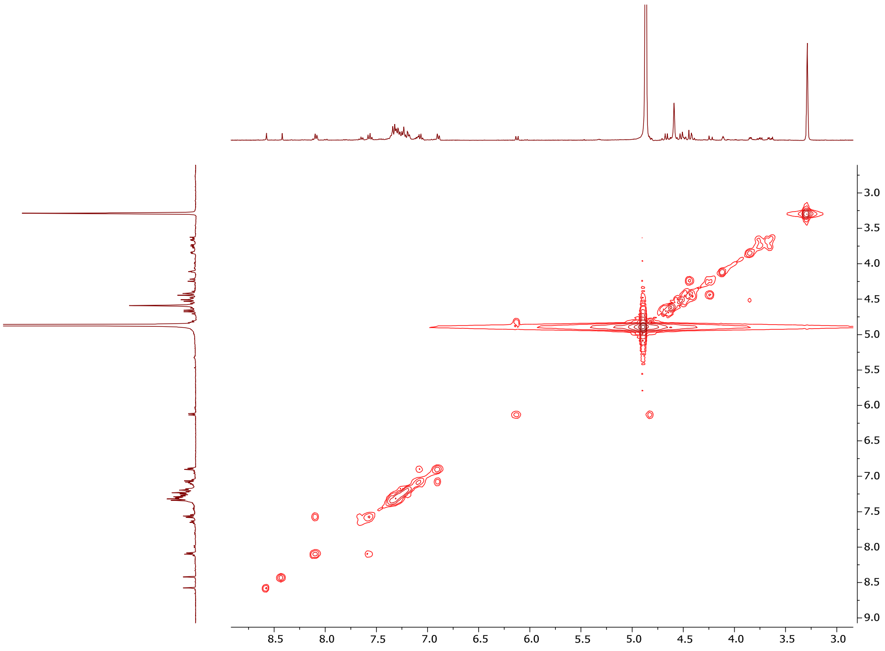

Figure S3. COSY spectrum for compound **5**.

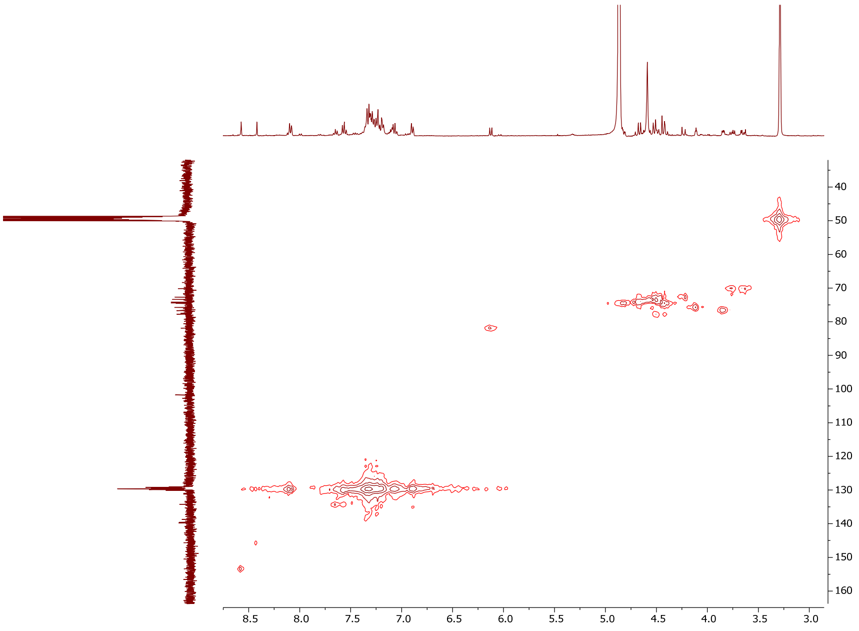

Figure S4. HSQC spectrum for compound **5**.

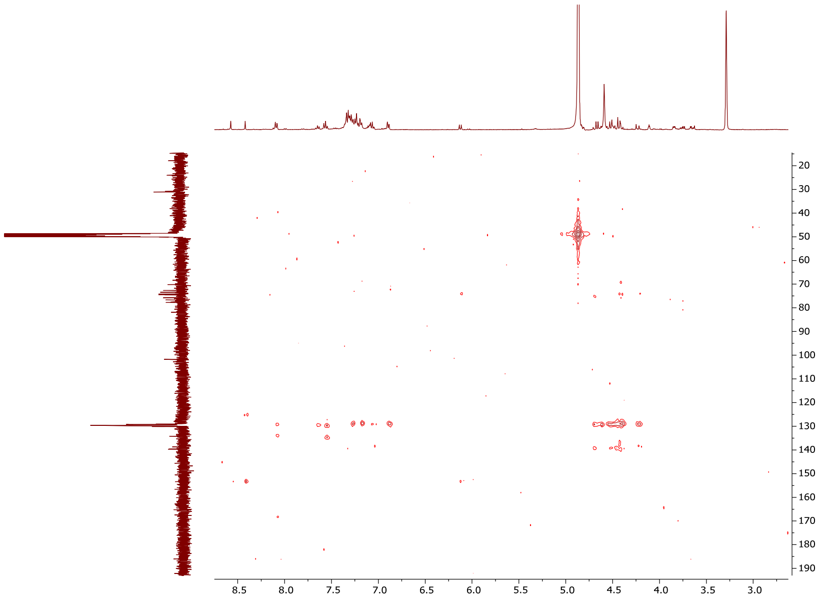

Figure S5. HMBC spectrum for compound **5**.

Compound **6** (in $CDCl_3$)

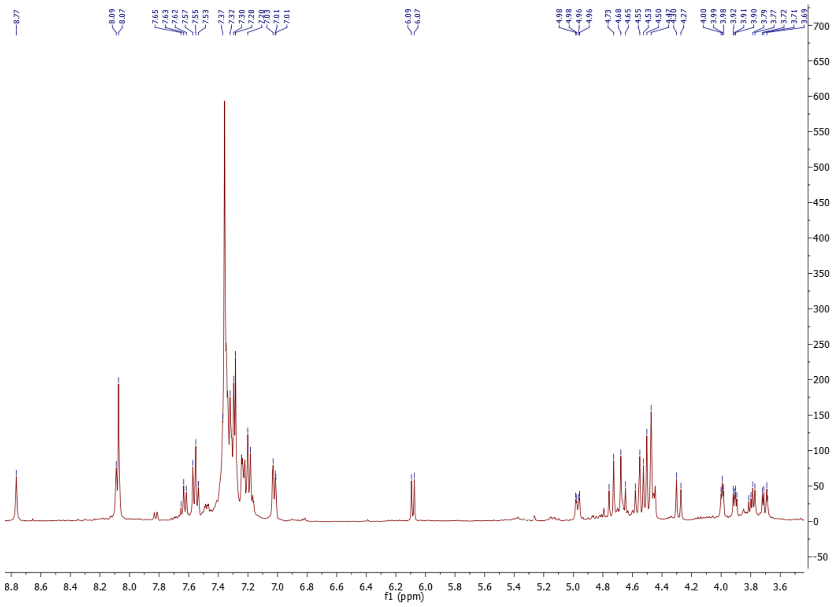

Figure S6. ^{1}H NMR spectrum for compound **6**.

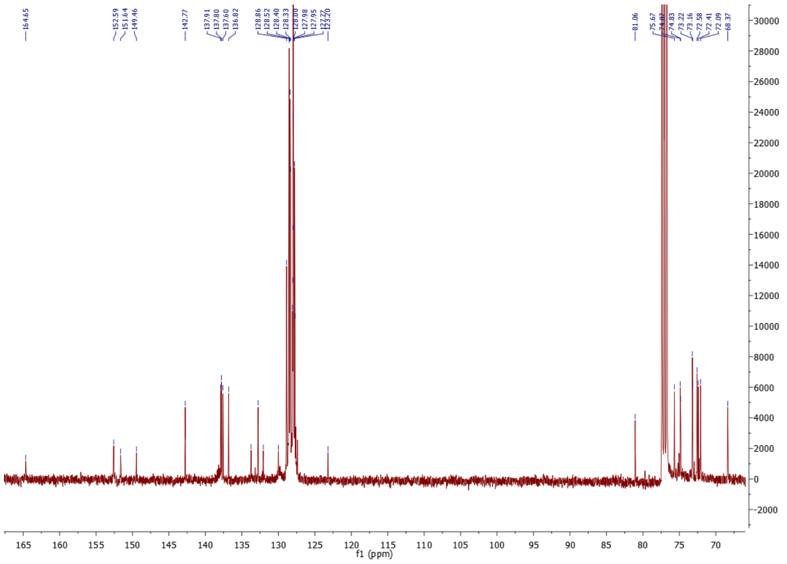

Figure S7. ^{13}C NMR spectrum for compound **6**.

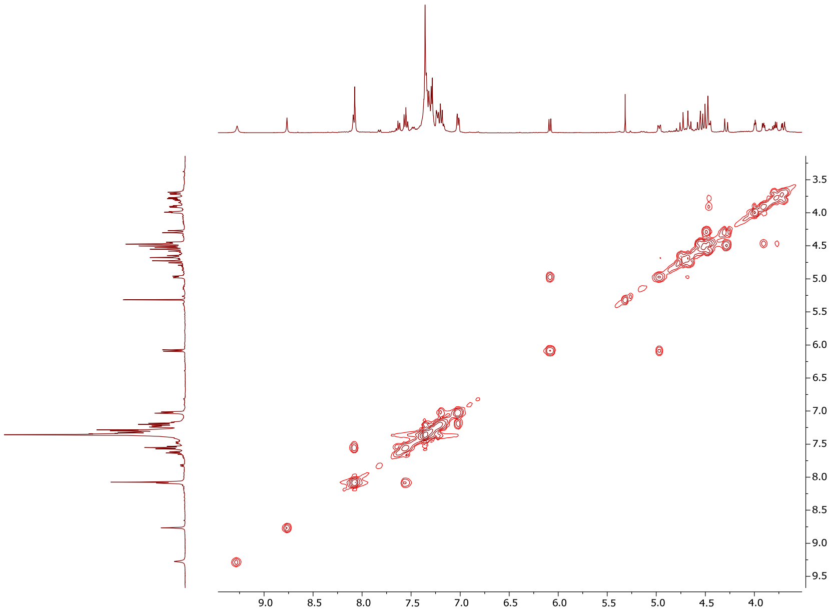

Figure S8. COSY spectrum for compound **6**.

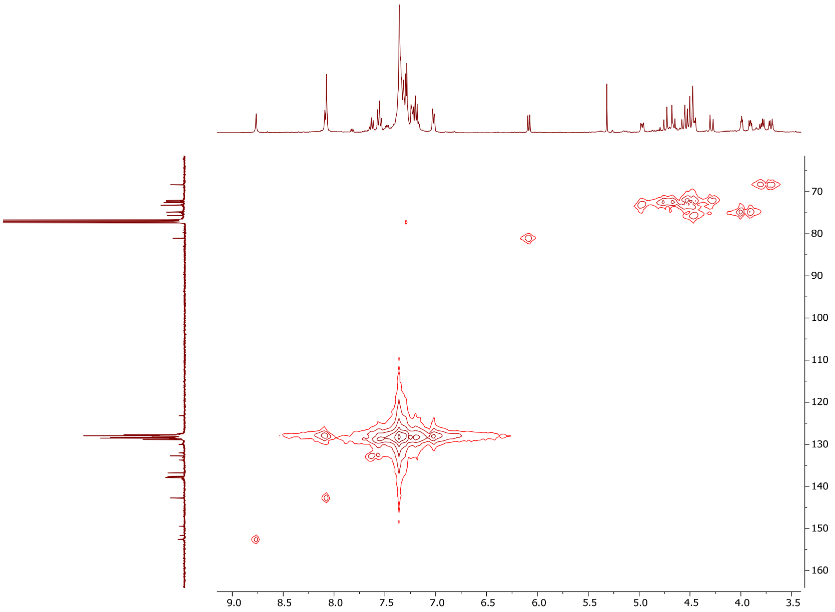

Figure S9. HSQC spectrum for compound **6**.

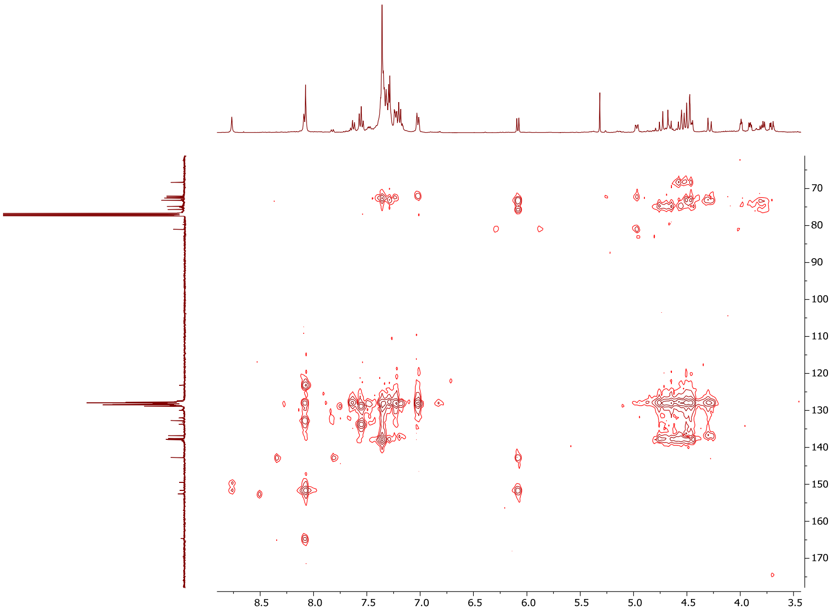

Figure S10. HMBC spectrum for compound **6**.

Compound **7** (in CD_3OD)

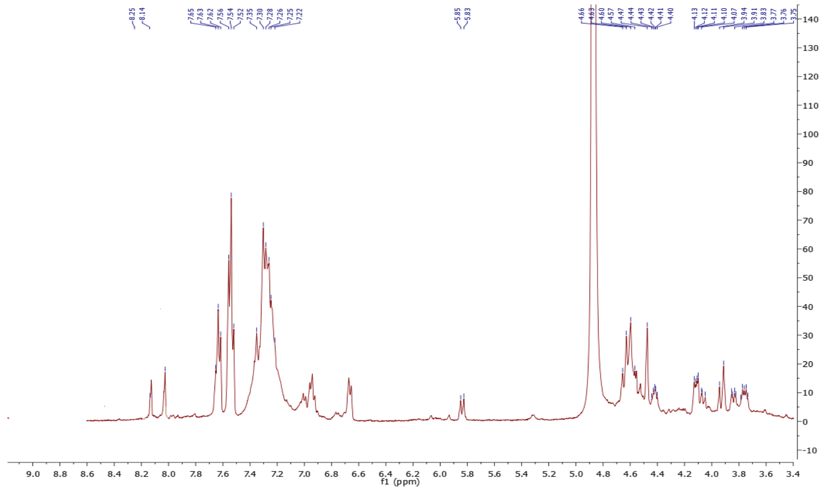

Figure S11. ^1H NMR spectrum for compound **7**.

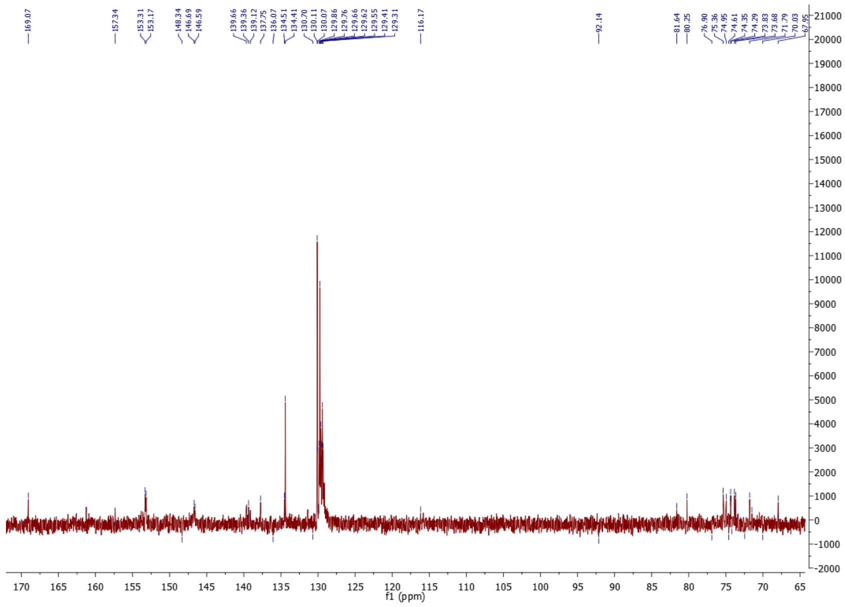

Figure S12. ^{13}C NMR spectrum for compound **7**.

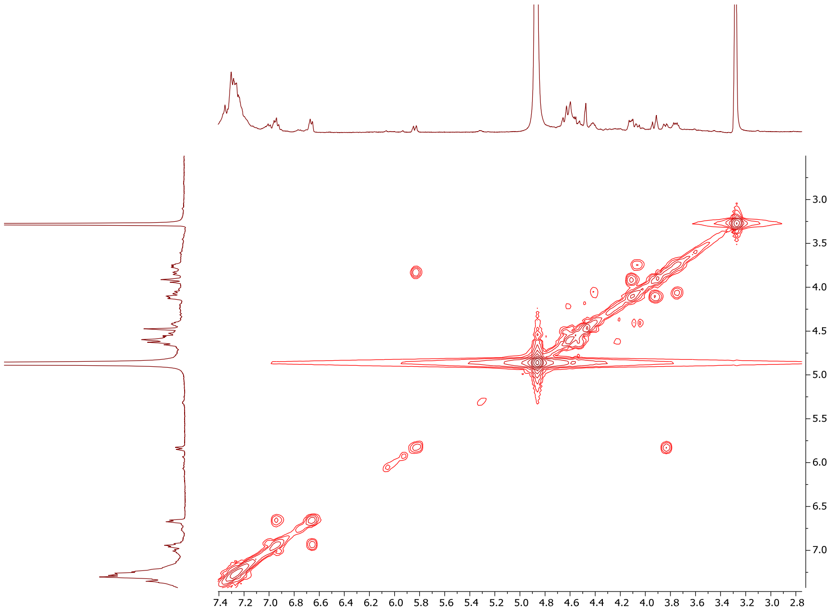

Figure S13. COSY spectrum for compound **7**.

Compound **8** (in CD_3OD)

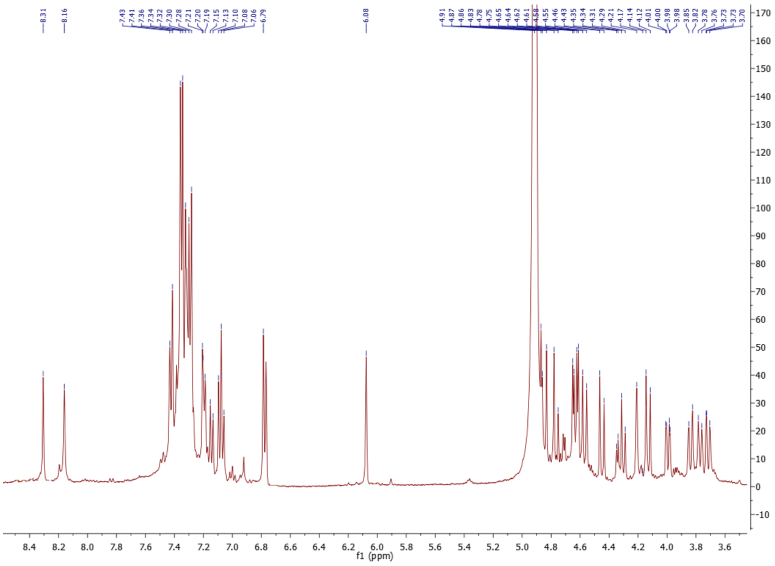

Figure S14. ^{1}H NMR spectrum for compound **8**.

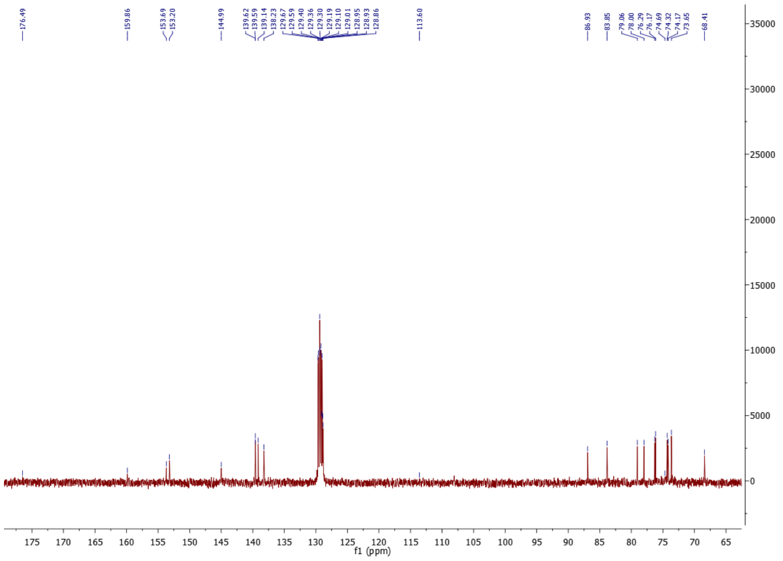

Figure S15. ^{13}C NMR spectrum for compound **8**.

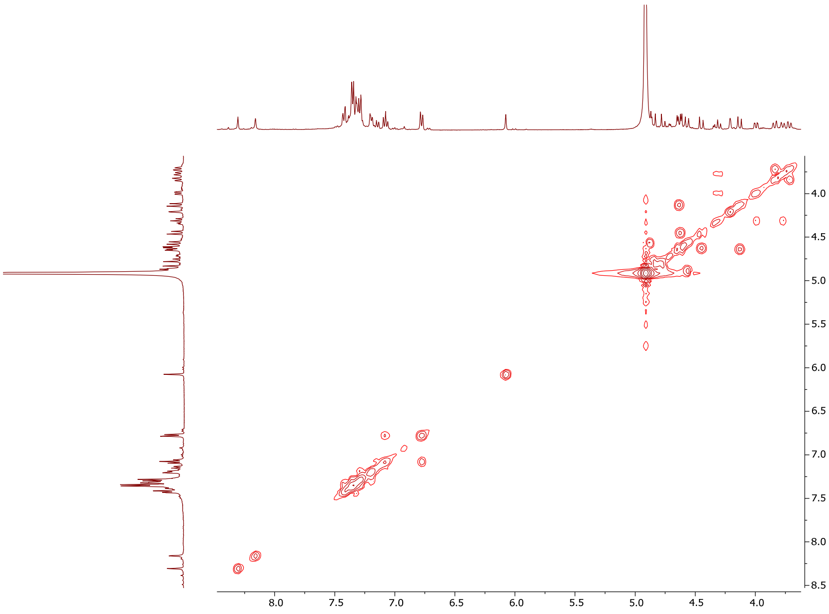

Figure S16. COSY spectrum for compound **8**.

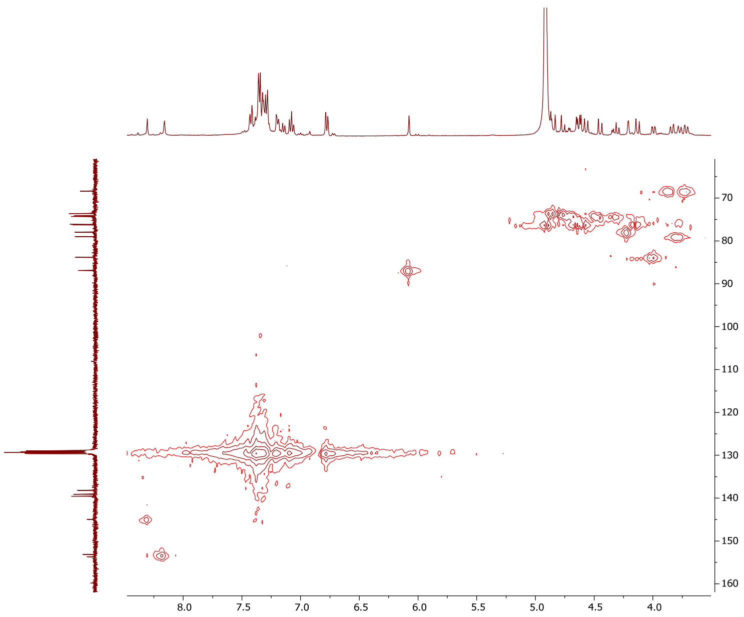

Figure S17. HSQC spectrum for compound **8**.

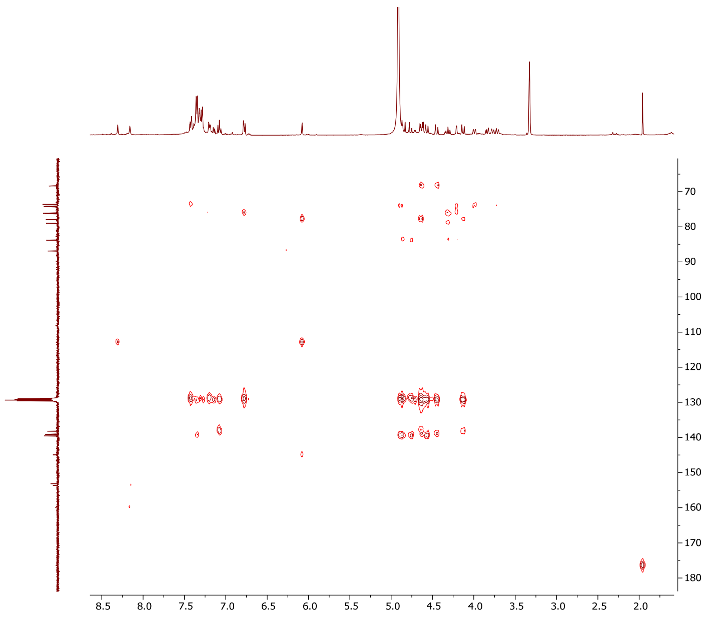

Figure S18. HMBC spectrum for compound **8**.

Compound **9** (in CD_3OD)

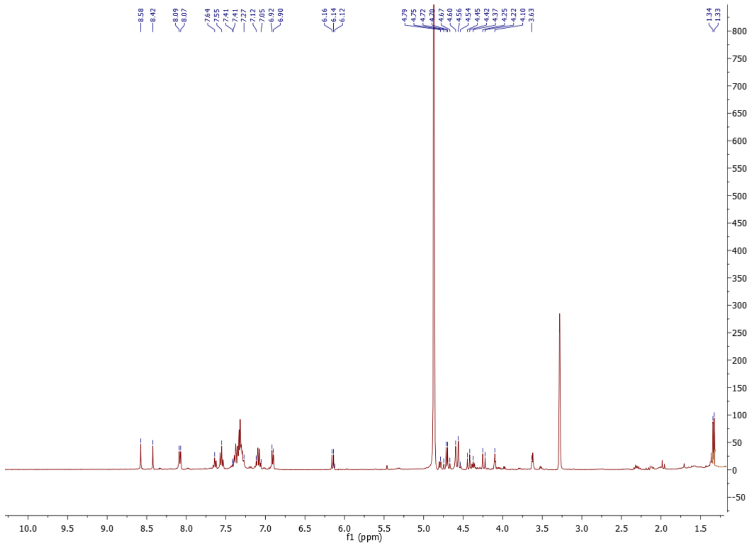

Figure S19. ^{1}H NMR spectrum for compound **9**.

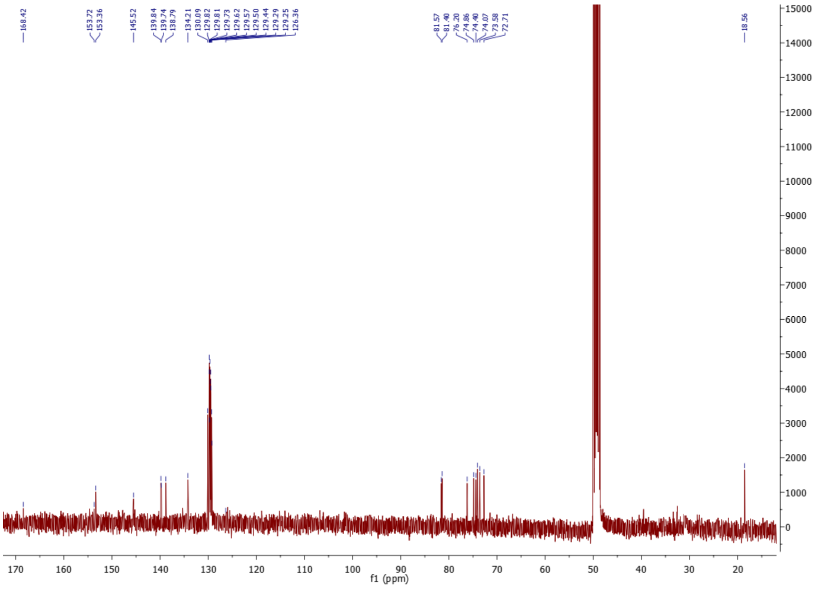

Figure S20. ^{13}C NMR spectrum for compound **9**.

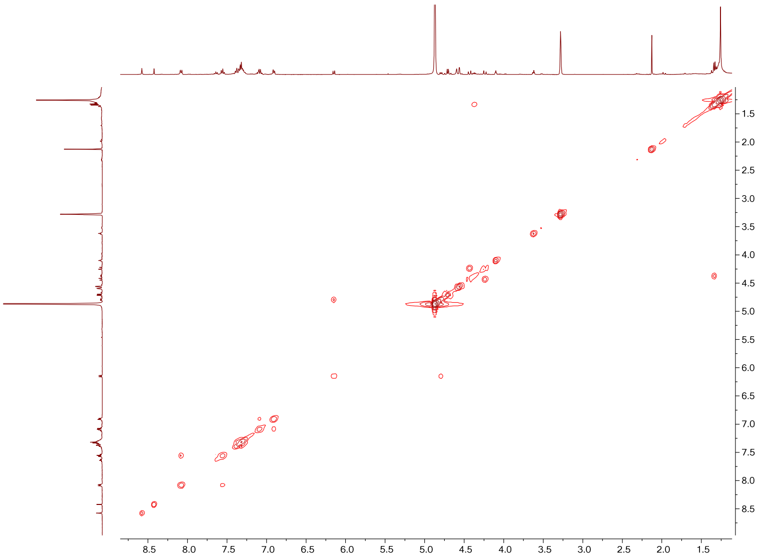

Figure S21. COSY spectrum for compound **9**.

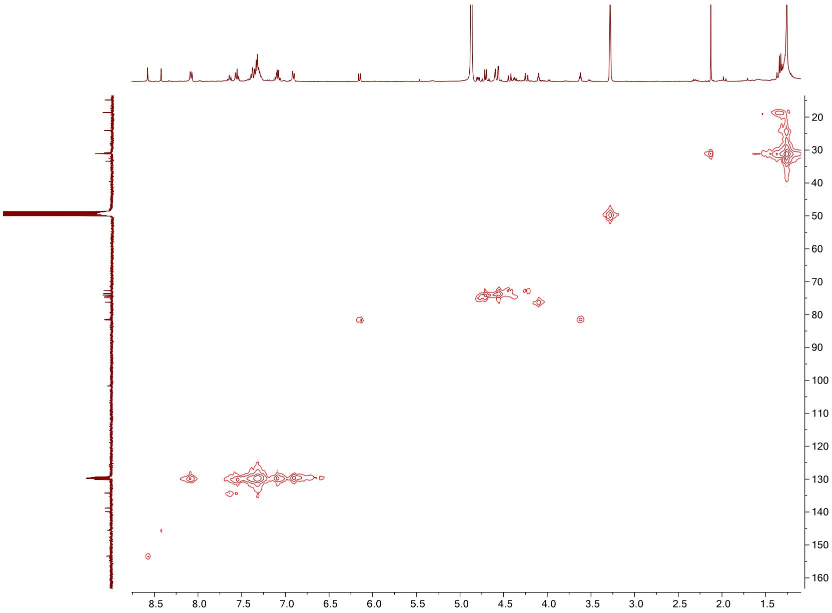

Figure S22. HSQC spectrum for compound **9**.

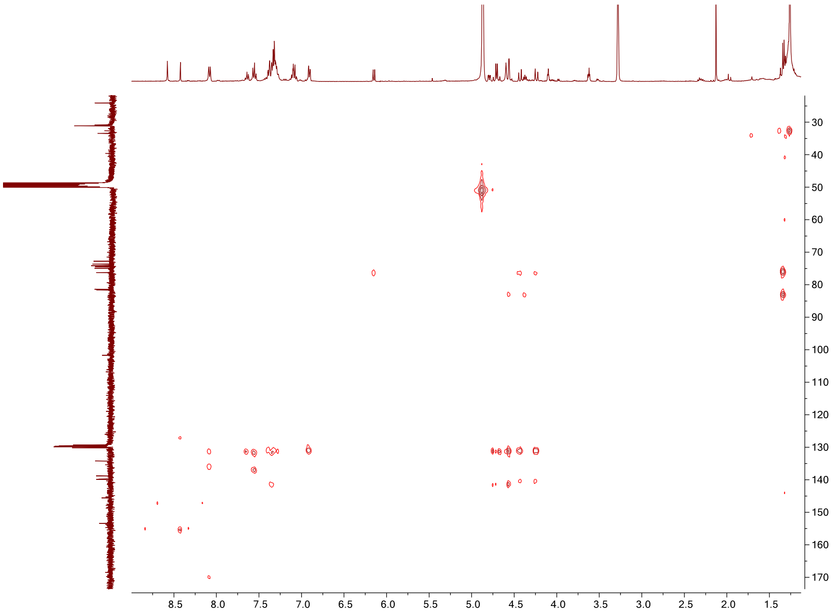

Figure S23. HMBC spectrum for compound **9**.

Compound **10** (in CD_3OD)

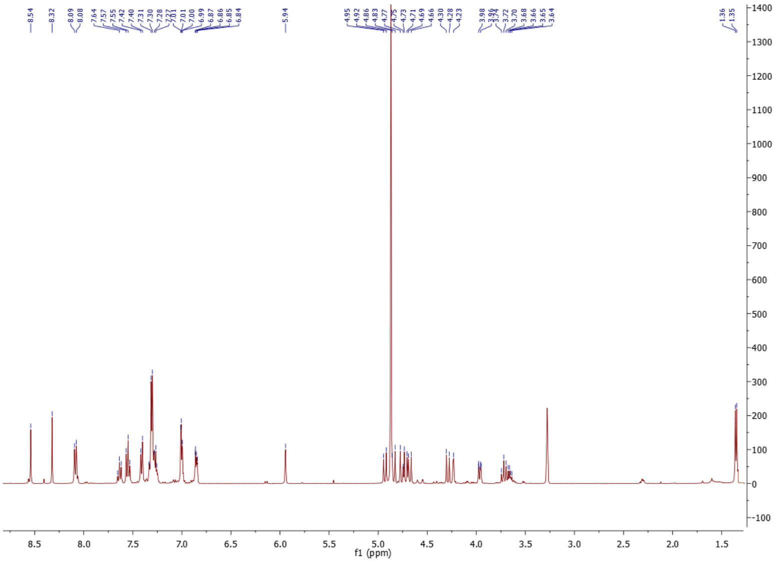

Figure S24. ^{1}H NMR spectrum for compound **10**.

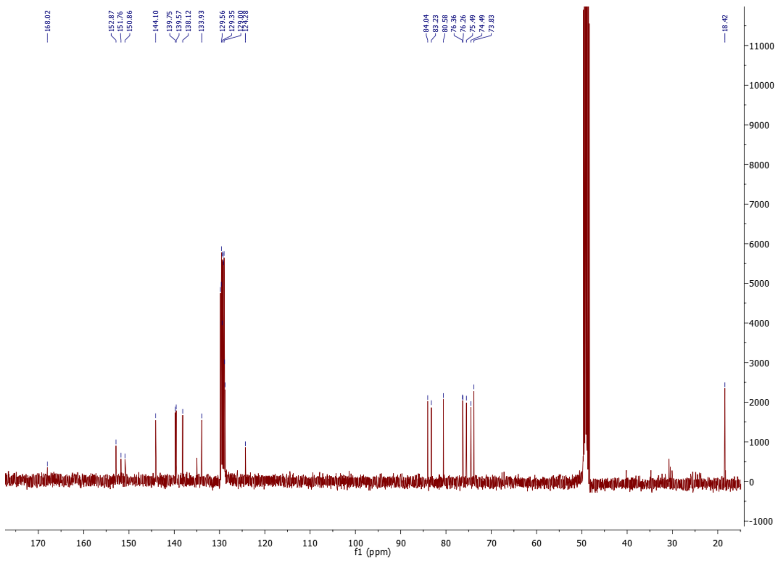

Figure S25. ^{13}C NMR spectrum for compound **10**.

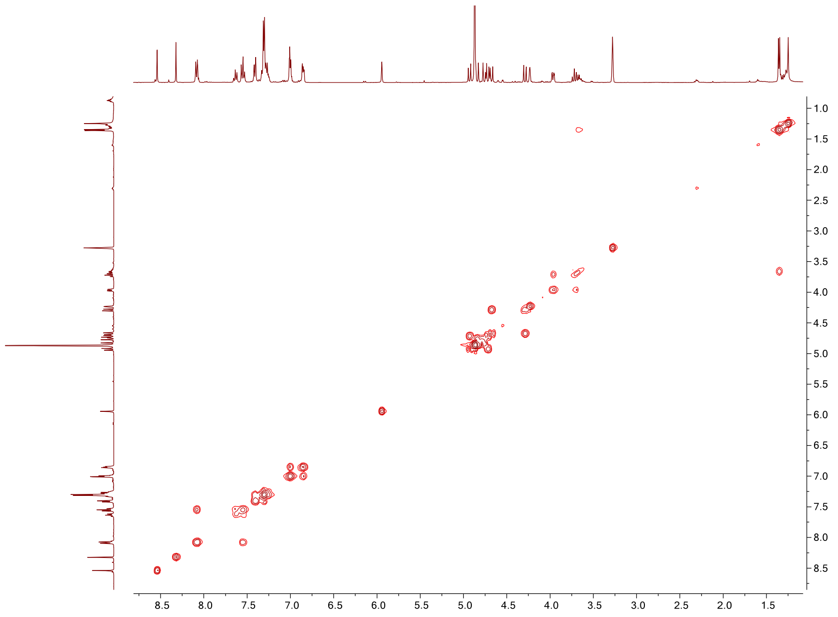

Figure S26. COSY spectrum for compound **10**.

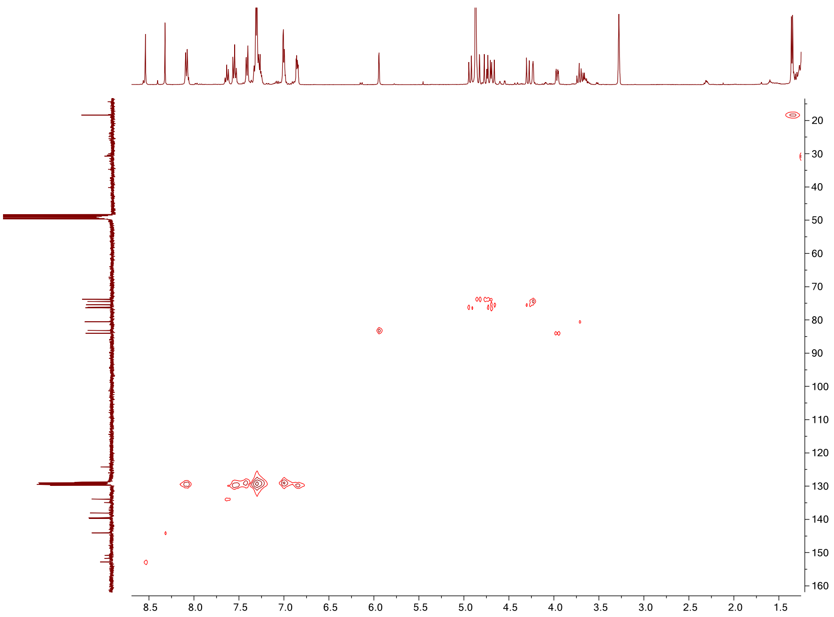

Figure S27. HSQC spectrum for compound **10**.

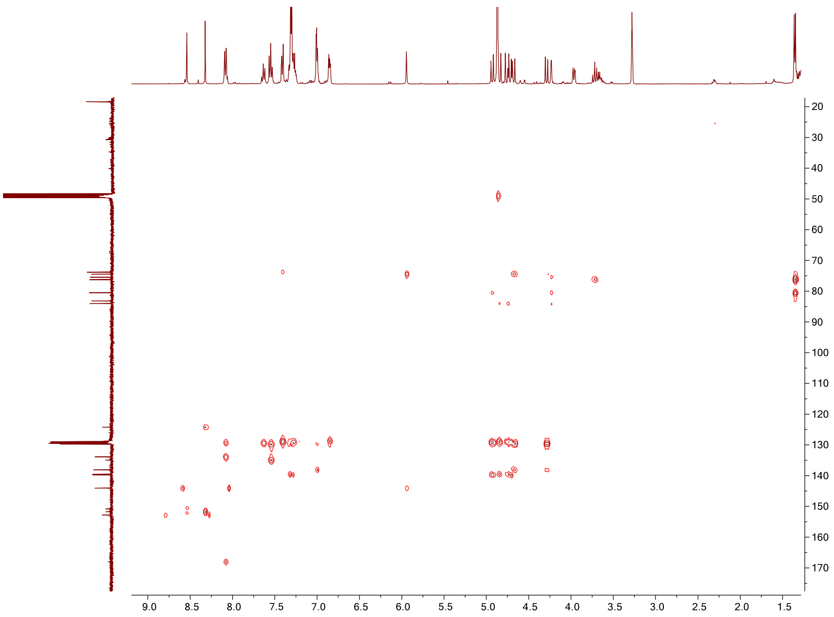

Figure S28. HMBC spectrum for compound **10**.

Compound **12** (in CD_3OD)

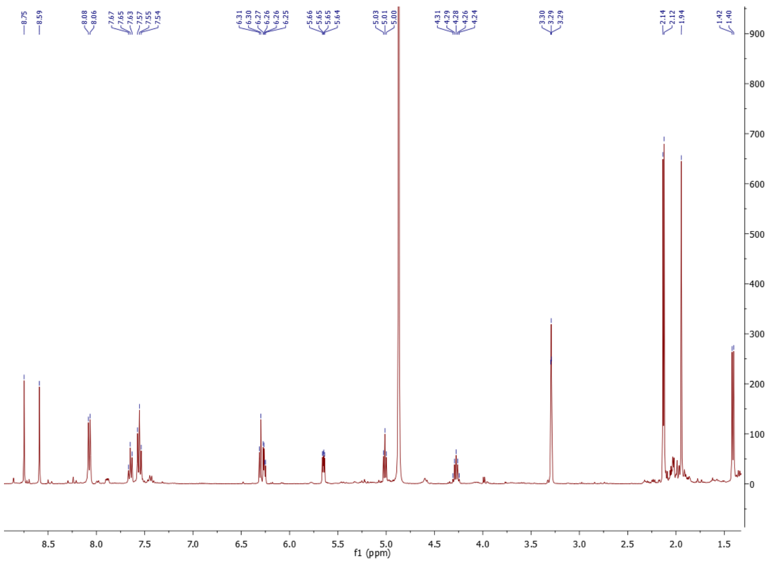

Figure S29. ^{1}H NMR spectrum for compound **12**.

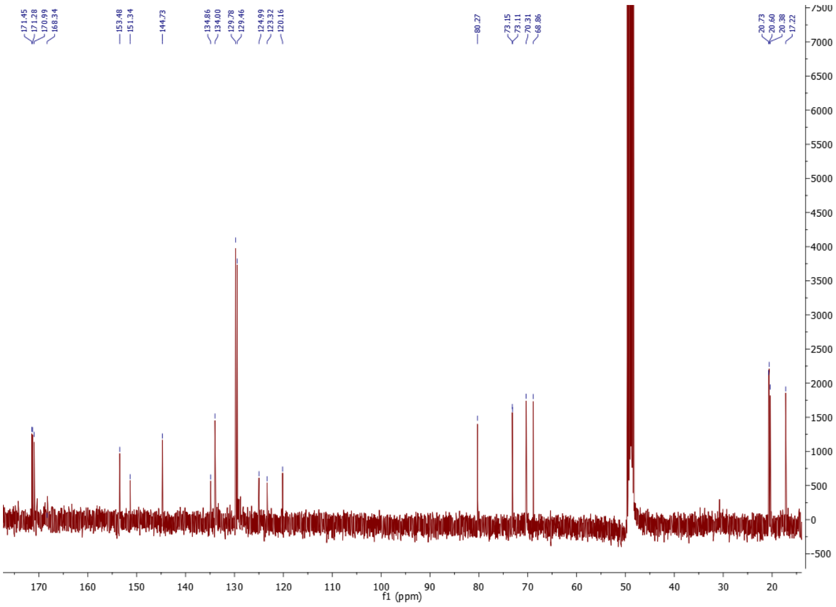

Figure S30. ^{13}C NMR spectrum for compound **12**.

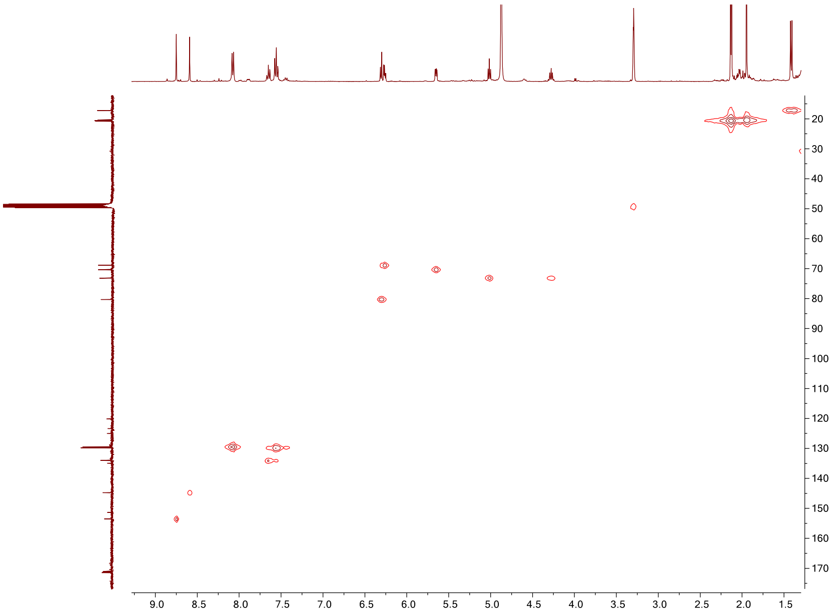

Figure S31. HSQC spectrum for compound **12**.

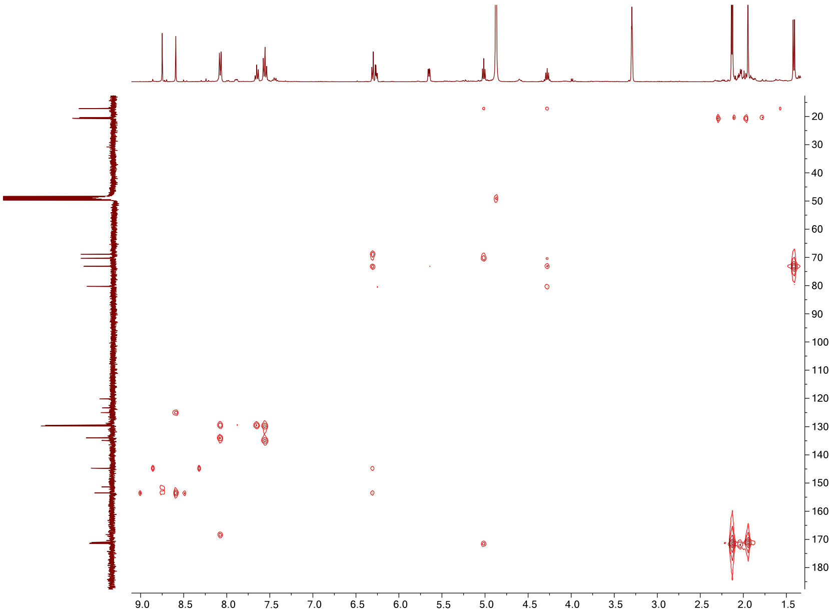

Figure S32. HMBC spectrum for compound **12**.

Compound **13** (in CD_3OD)

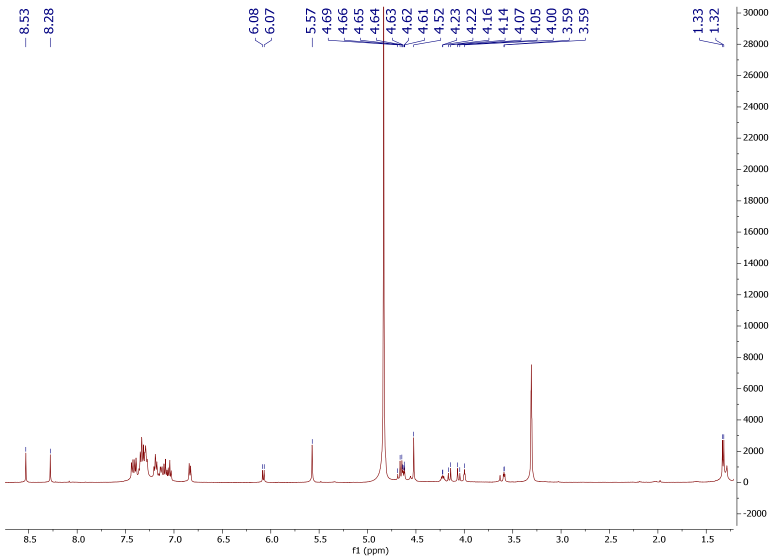

Figure S33. ^{1}H NMR spectrum for compound **13**.

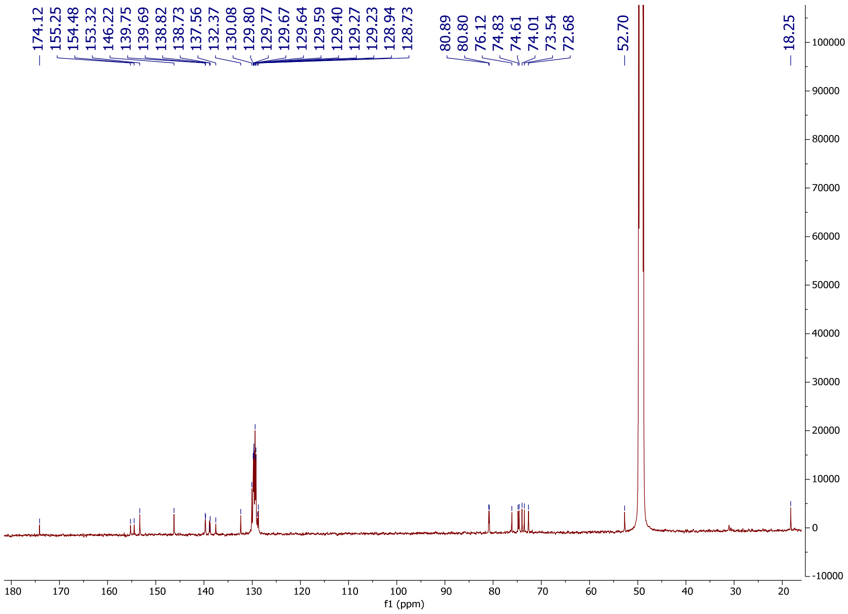

Figure S34. ^{13}C NMR spectrum for compound **13**.

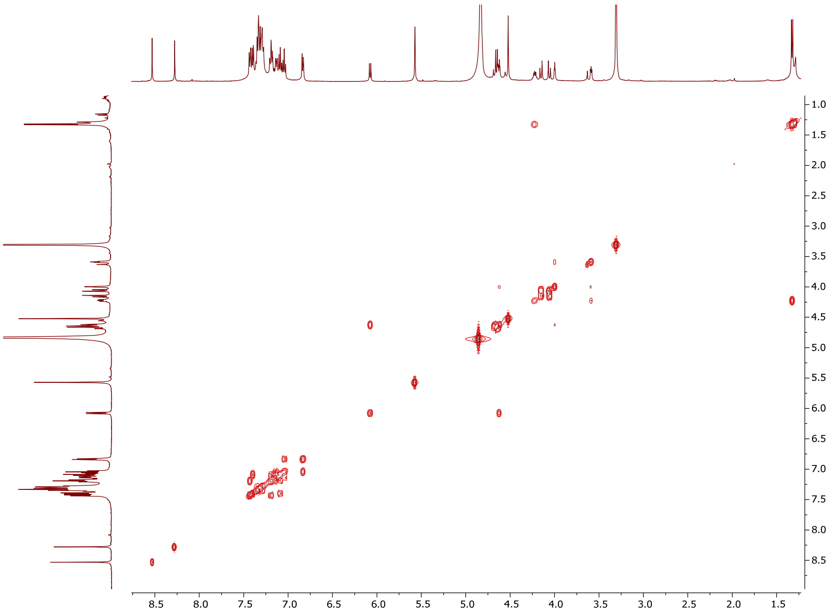

Figure S35. COSY spectrum for compound **13**.

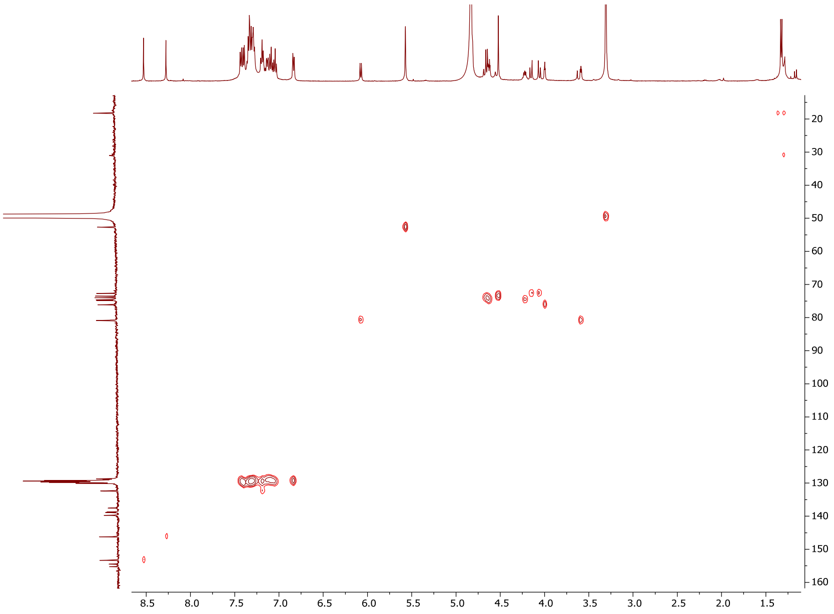

Figure S36. HSQC spectrum for compound **13**.

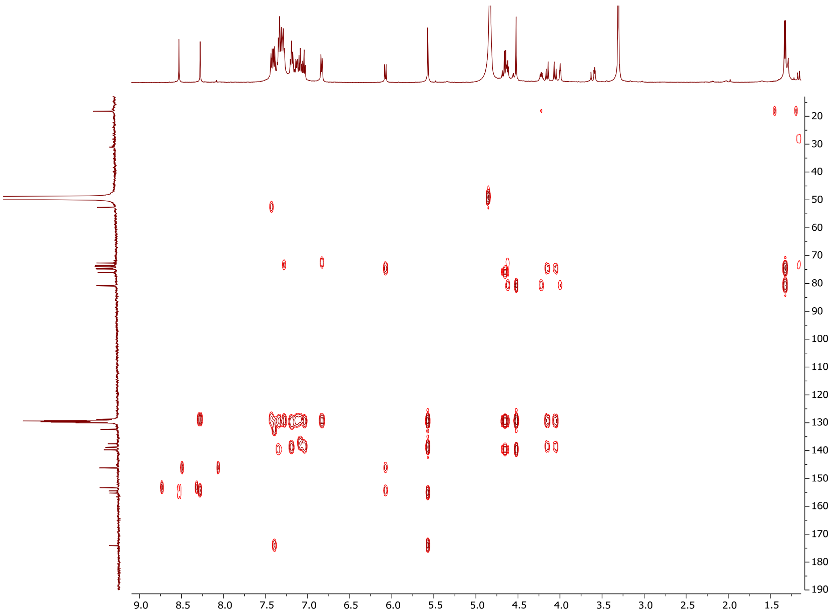

Figure S37. HMBC spectrum for compound **13**.

Compound **15** (in CD_3OD)

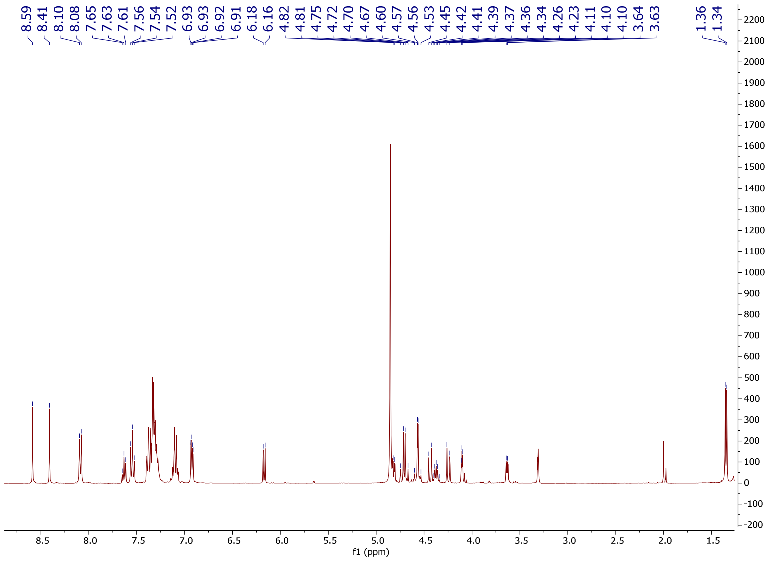

Figure S38. ^{1}H NMR spectrum for compound **15**.

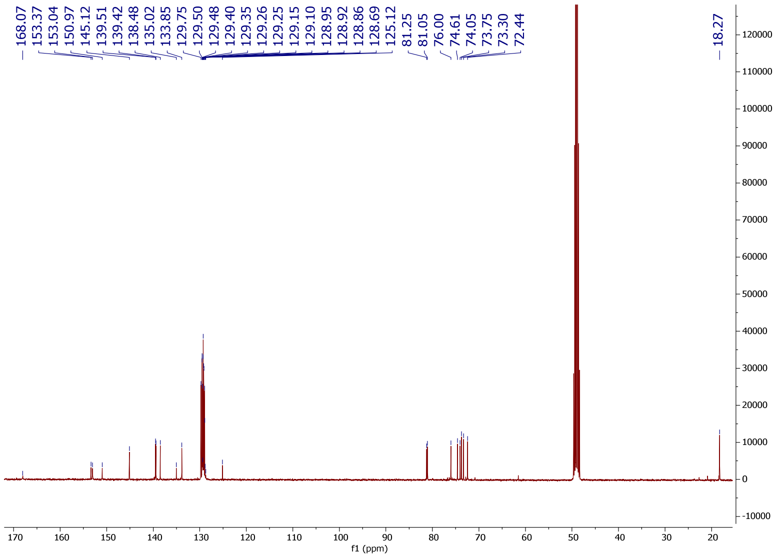

Figure S39. ^{13}C NMR spectrum for compound **15**.

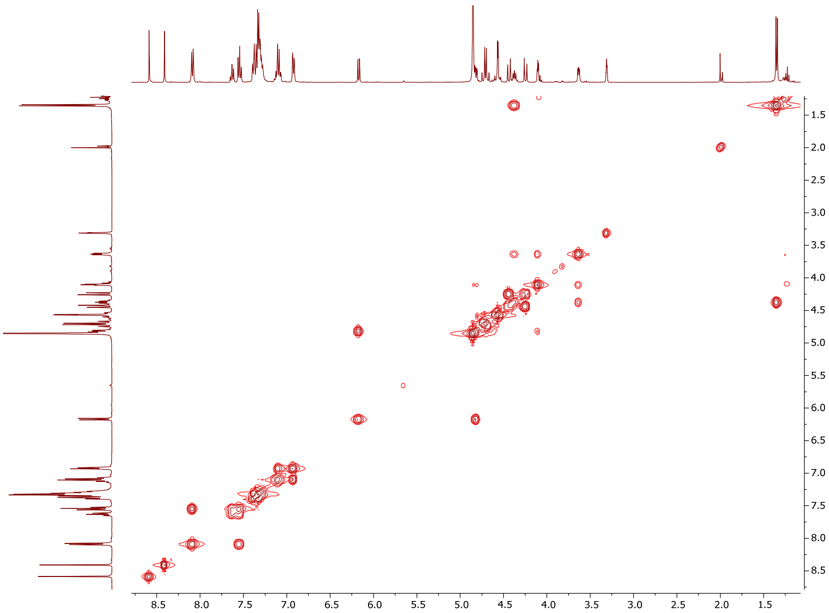

Figure S40. COSY spectrum for compound **15**.

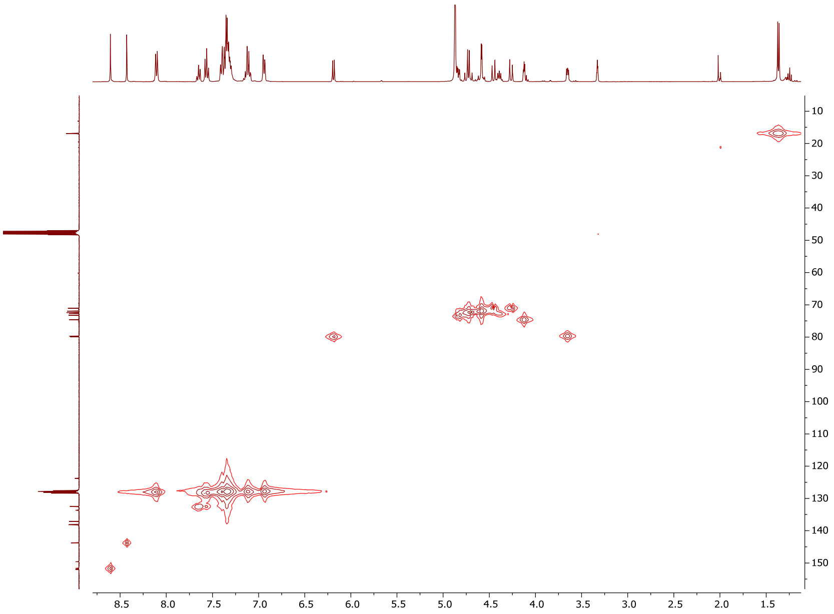

Figure S41. HSQC spectrum for compound **15**.

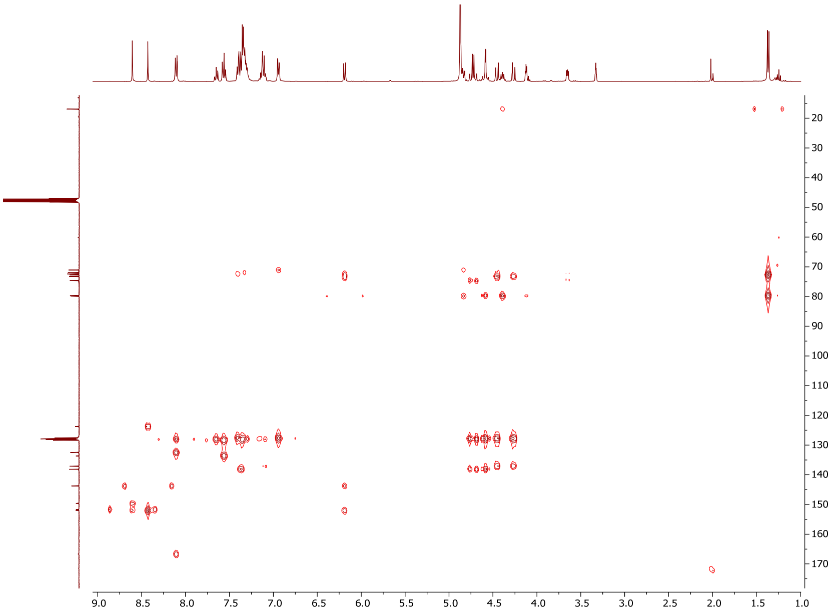

Figure S42. HMBC spectrum for compound **15**.

Compound **16** (in CD_3OD)

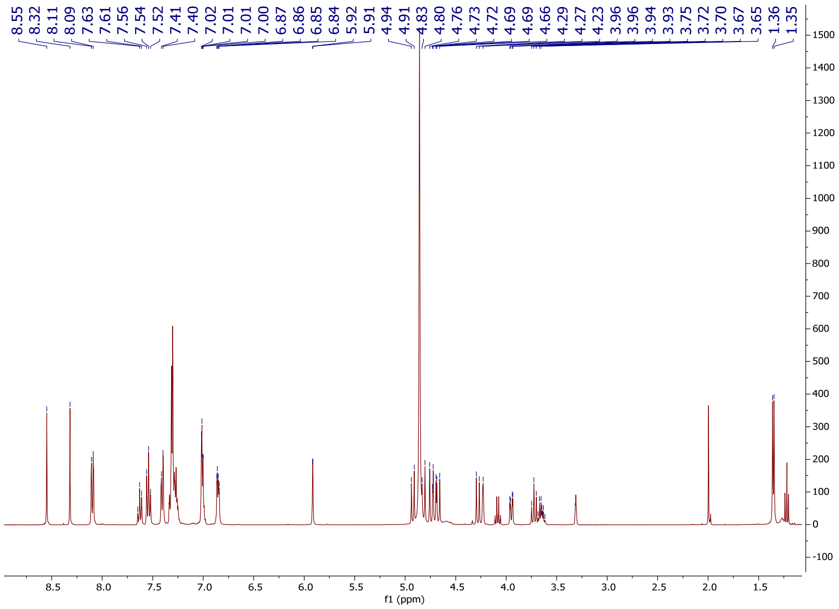

Figure S43. ^{1}H NMR spectrum for compound **16**.

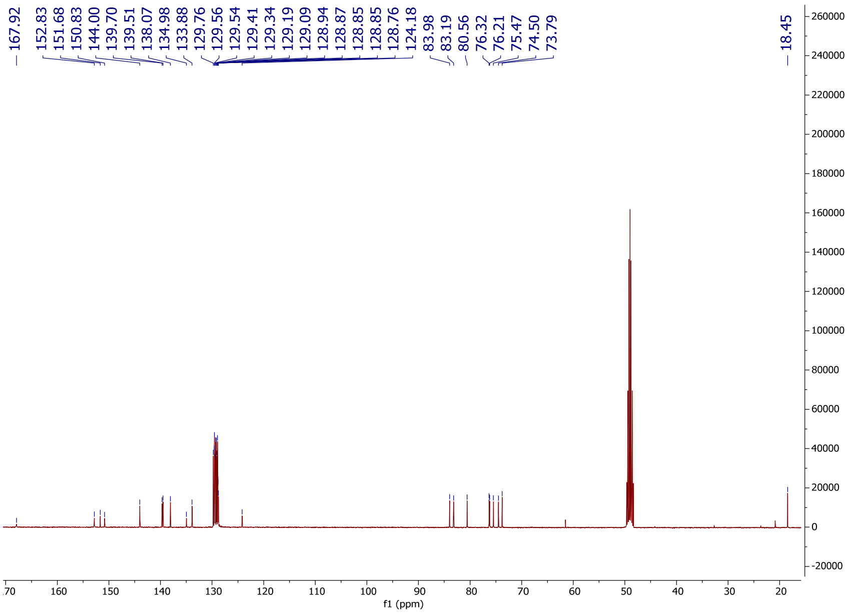

Figure S44. ^{13}C NMR spectrum for compound **16**.

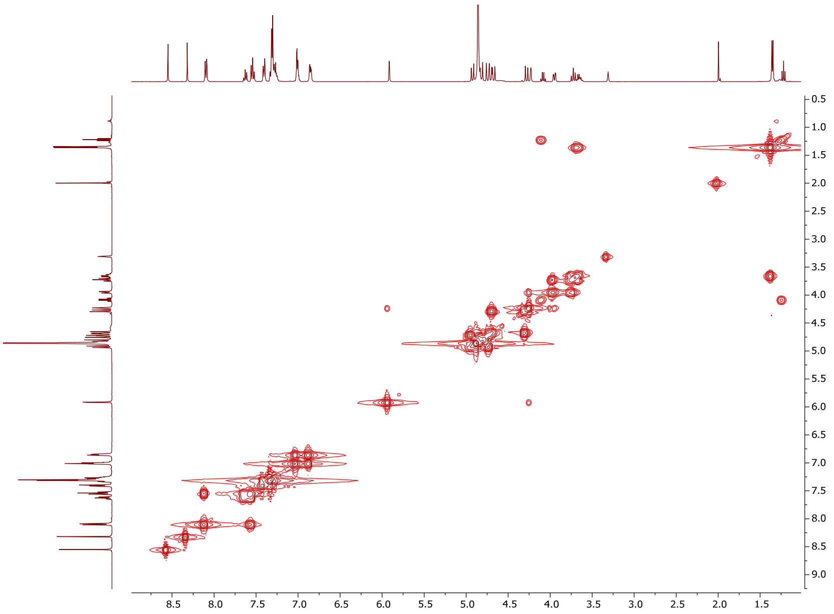

Figure S45. COSY spectrum for compound **16**.

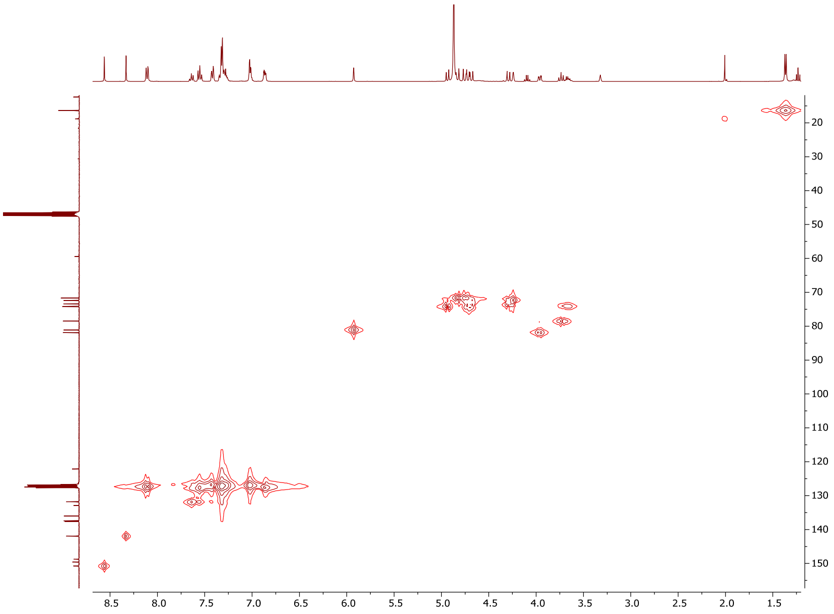

Figure S46. HSQC spectrum for compound **16**.

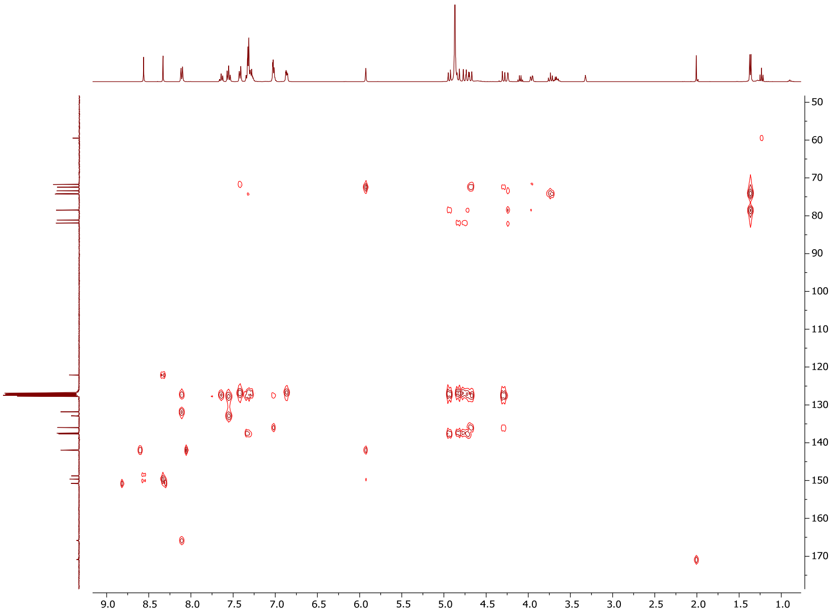

Figure S47. HMBC spectrum for compound **16**.

^{1}H NMR and ^{13}C NMR spectra by computational calculation

Table S1. NMR chemical shifts calculated for compound **5**.

H (paper)	^{1}H δ calc	^{1}H δ exp	C (paper)	^{13}C δ calc	^{13}C δ exp
H-2	8.36	8.58	Ph	126.8	130.1-129.0
H-8	7.89	8.42	Ph	126.8	130.1-129.0
NH	8.70	7.36-6.89	Ph	129.1	130.1-129.0
ortho PhCO	7.90	8.09	Ph-Cq	137.6	139.7
ortho PhCO	8.03	8.09	Ph	129.2	130.1-129.0
meta PhCO	7.54	7.57	Ph	126.8	130.1-129.0
meta PhCO	7.56	7.57	CH_2Ph	72.6	74.6
para PhCO	7.65	7.65	C-6'	67.4	70.2
H-4'	3.93	3.85	C-5'	73.2	74.1
H-3'	4.39	4.11	C-1'	73.8	81.9
H-5'	4.26	4.60-4.43	C-2'	76.8	74.4
H-2'	3.98	4.78	C-3'	74.4	75.8
H-1'	5.99	6.13	C-4'	74.5	76.7
H-6'b	4.06	3.76	CH_2Ph	70.8	73.4
H-6'a	3.46	3.65	Ph-Cq	137.2	139.6
CH_2Ph-2'	3.97	4.25, 4.22	Ph	129.4	130.1-129.0
CH_2Ph-2'	4.56	4.60 -4.43	Ph	126.9	130.1-129.0
CH_2Ph-4'	4.09	4.60-4.43	Ph	126.9	130.1-129.0
CH_2Ph-4'	4.30	4.60-4.43	Ph	126.8	130.1-129.0
CH_2Ph-3'	4.57	4.71, 4.68, 4.66, 4.63	Ph	129.1	130.1-129.0
CH_2Ph-3'	4.83	4.71, 4.68, 4.66, 4.63	CH_2Ph	74.9	77.7
CH_2Ph-6'	3.80	4.60-4.43	Ph-Cq	137.8	139.7
CH_2Ph-6'	4.10	4.60-4.43	Ph	129.9	130.1-129.0
Ph-2'	6.50	7.36-6.89	Ph	127.3	130.1-129.0
Ph-2'	7.26	7.36-6.89	Ph	127.0	130.1-129.0
Ph-2'	7.02	7.36-6.89	Ph	126.7	130.1-129.0

Ph-2'	7.23	7.36-6.89	Ph	128.8	130.1-129.0
Ph-2'	7.19	7.36-6.89	CH_2Ph	70.4	72.7
Ph-3'	7.76	7.36-6.89	Ph-Cq	136.7	138.7
Ph-3'	7.17	7.36-6.89	Ph	128.8	130.1-129.0
Ph-3'	7.64	7.36-6.89	Ph	126.7	130.1-129.0
Ph-3'	7.34	7.36-6.89	Ph	126.7	130.1-129.0
Ph-3'	7.42	7.36-6.89	Ph	126.5	130.1-129.0
Ph-4'	7.48	7.36-6.89	Ph	128.0	130.1-129.0
Ph-4'	7.40	7.36-6.89	C-4	148.9	156.6
Ph-4'	7.41	7.36-6.89	C-5	119.3	127.9
Ph-4'	7.40	7.36-6.89	C-8	139.1	145.6
Ph-4'	7.38	7.36-6.89	C-6	147.6	150.5
Ph-6'	7.54	7.36-6.89	C-2	149.8	153.4
Ph-6'	7.37	7.36-6.89	CONH	162.9	167.4
Ph-6'	7.49	7.36-6.89	Ph-Cq	133.8	134.4
Ph-6'	7.37	7.36-6.89	Ph	124.9	130.1-129.0
Ph-6'	7.38	7.36-6.89	Ph	126.9	130.1-129.0
			Ph	131.9	134.3
			Ph	127.5	130.1-129.0
			Ph	128.2	130.1-129.0

Table S2. NMR chemical shifts calculated for compound **6**.

H (paper)	^{1}H δ calc	^{1}H δ exp	C (paper)	^{13}C δ calc	^{13}C δ exp
H-2	8.49	8.77	C-4	147.6	151.6
H-8	8.14	8.09	C-5	118.8	123.2
NH	8.72	7.37-7.01	C-6	147.4	149.6
ortho PhCO	7.85	8.08	C-2	149.6	152.6
ortho PhCO	8.05	8.08	C-8	141.5	142.8
meta PhCO	7.52	7.55	CONH	162.7	164.6
meta PhCO	7.56	7.55	Ph-Cq	133.6	136.8
para PhCO	7.64	7.63	Ph	124.8	127.8-128.9

H-4'	4.35	3.91	Ph	128.2	127.8-128.9
H-3'	3.86	3.99	Ph	126.9	127.8-128.9
H-5'	3.43	4.55-4.43	Ph	127.5	127.8-128.9
H-2'	4.10	4.97	Ph	131.9	127.8-128.9
H-1'	5.87	6.08	C-4'	70.7	74.9, 74.8
H-6'b	3.67	3.71	C-3'	82.9	74.9, 74.8
H-6'a	4.22	3.78	C-5'	80.2	75.7
CH_2Ph-2'	4.84	4.55-4.43	C-2'	72.7	73.2
CH_2Ph-2'	3.96	4.55-4.43	C-1'	78.1	81.1
CH_2Ph-3'	4.78	4.68, 4.65	C-6'	68.0	68.4
CH_2Ph-3'	5.16	4.76, 4.73	CH_2Ph	74.2	73.2
CH_2Ph-4'	4.87	4.55-4.43	CH_2Ph	67.9	72.1
CH_2Ph-4'	4.37	4.55-4.43	CH_2Ph	73.8	72.4
CH_2Ph-6'	4.67	4.55-4.43	CH_2Ph	74.1	72.4
CH_2Ph-6'	4.08	4.30, 4.27	Ph-Cq	137.3	137.5
Ph-2'	7.84	7.37-7.01	Ph	129.9	127.8-128.9
Ph-2'	7.31	7.37-7.01	Ph	129.4	127.8-128.9
Ph-2'	7.69	7.37-7.01	Ph	127.4	127.8-128.9
Ph-2'	7.42	7.37-7.01	Ph	127.0	127.8-128.9
Ph-2'	7.51	7.37-7.01	Ph	127.2	127.8-128.9
Ph-3'	8.00	7.37-7.01	Ph-Cq	139.2	137.9
Ph-3'	7.26	7.37-7.01	Ph	122.5	127.8-128.9
Ph-3'	7.49	7.37-7.01	Ph	123.4	127.8-128.9
Ph-3'	7.38	7.37-7.01	Ph	127.1	127.8-128.9
Ph-3'	7.25	7.37-7.01	Ph	126.5	127.8-128.9
Ph-4'	7.10	7.37-7.01	Ph	124.4	127.8-128.9
Ph-4'	7.29	7.37-7.01	Ph-Cq	137.7	137.5
Ph-4'	7.17	7.37-7.01	Ph	129.0	127.8-128.9
Ph-4'	7.29	7.37-7.01	Ph	129.1	127.8-128.9
Ph-4'	7.25	7.37-7.01	Ph	126.6	127.8-128.9
Ph-6'	6.71	7.37-7.01	Ph	126.7	127.8-128.9
Ph-6'	7.41	7.37-7.01	Ph	126.6	127.8-128.9

Ph-6'	7.26	7.37-7.01	Ph-Cq	138.3	137.7
Ph-6'	7.38	7.37-7.01	Ph	128.6	127.8-128.9
Ph-6'	7.34	7.37-7.01	Ph	129.2	127.8-128.9
			Ph	126.4	127.8-128.9
			Ph	126.8	127.8-128.9
			Ph	126.5	127.8-128.9

Table S3. NMR chemical shifts calculated for compound **7**.

H (paper)	^{1}H δ calc	^{1}H δ exp	C (paper)	^{13}C δ calc	^{13}C δ exp
H-2	8.61	8.25	C-4	159.7	157.3
H-8	8.09	8.14	C-5	117.2	116.2
NH	7.46	7.35-6.60	C-6	141.7	153.2
ortho PhCO	7.68	7.35-6.60	C-2	149.3	153.3
ortho PhCO	7.66	7.35-6.60	C-8	145.6	146.7
meta PhCO	7.35	7.35-6.60	CONH	164.8	169.1
meta PhCO	7.10	7.35-6.60	Ph-Cq	132.8	136.1
para PhCO	7.38	7.35-6.60	Ph	124.9	129.9-129.3
H-4'	3.94	4.12-4.06	Ph	128.0	129.9-129.3
H-3'	4.18	4.66-4.40	Ph	126.6	129.9-129.3
H-5'	4.39	3.91	Ph	127.4	129.9-129.3
H-2'	3.81	3.85	Ph	131.6	134.5
H-1'	5.94	5.84	C-4'	74.2	73.7
H-6'a	4.18	4.12-4.06	C-3'	75.0	80.3
H-6'b	3.64	3.75	C-5'	73.7	71.8
CH_2Ph-2'	4.01	4.66-4.40	C-2'	77.5	74.4
CH_2Ph-2'	4.80	4.66-4.40	C-1'	76.5	81.6
CH_2Ph-4'	4.07	4.66-4.40	C-6'	67.5	67.9
CH_2Ph-4'	4.32	4.66-4.40	CH_2Ph	67.3	73.8
CH_2Ph-3'	4.39	4.66-4.40	CH_2Ph	70.9	74.3
CH_2Ph-3'	4.61	4.66-4.40	CH_2Ph	75.1	75.4
CH_2Ph-6'	4.15	4.66-4.40	CH_2Ph	72.9	75.0

CH_2Ph-6'	4.30	4.66-4.40	Ph-Cq	138.0	139.1
Ph-2'	7.00	7.35-6.60	Ph	121.8	129.9-129.3
Ph-2'	6.96	7.35-6.60	Ph	123.0	129.9-129.3
Ph-2'	7.21	7.35-6.60	Ph	126.7	129.9-129.3
Ph-2'	7.21	7.35-6.60	Ph	126.2	129.9-129.3
Ph-2'	7.12	7.35-6.60	Ph	124.3	129.9-129.3
Ph-3'	7.48	7.35-6.60	Ph-Cq	138.7	139.7
Ph-3'	6.80	7.35-6.60	Ph	124.8	129.9-129.3
Ph-3'	7.40	7.35-6.60	Ph	124.8	129.9-129.3
Ph-3'	7.29	7.35-6.60	Ph	126.8	129.9-129.3
Ph-3'	7.26	7.35-6.60	Ph	126.7	129.9-129.3
Ph-4'	7.45	7.35-6.60	Ph	125.8	129.9-129.3
Ph-4'	7.42	7.35-6.60	Ph-Cq	137.1	137.8
Ph-4'	7.40	7.35-6.60	Ph	129.5	134.4
Ph-4'	7.41	7.35-6.60	Ph	129.0	130.1
Ph-4'	7.38	7.35-6.60	Ph	126.9	129.9-129.3
Ph-6'	7.55	7.35-6.60	Ph	126.8	129.9-129.3
Ph-6'	7.49	7.35-6.60	Ph	126.9	129.9-129.3
Ph-6'	7.41	7.35-6.60	Ph-Cq	137.8	139.4
Ph-6'	7.41	7.35-6.60	Ph	128.6	130.0
Ph-6'	7.37	7.35-6.60	Ph	129.4	130.7
			Ph	126.7	129.9-129.3
			Ph	126.9	129.9-129.3
			Ph	126.8	129.9-129.3

Table S4. NMR chemical shifts calculated for compound **9**.

H (paper)	^{1}H δ calc	^{1}H δ exp	C (paper)	^{13}C δ calc	^{13}C δ exp
H-2	8.35	8.58	C-4	148.8	153.7
H-8	7.90	8.42	C-5	119.2	126.4
NH	8.73	7.40-7.27	C-6	147.5	153.4
ortho PhCO	7.91	8.08	C-2	149.7	153.4

ortho PhCO	8.04	8.08	C-8	139.2	145.5
meta PhCO	7.54	7.55	CONH	162.8	168.4
meta PhCO	7.56	7.55	Ph-Cq	133.7	134.2
para PhCO	7.64	7.64	Ph	124.8	129.2-130.1
H-4'	3.69	3.62	Ph	128.2	129.2-130.1
H-3'	4.33	4.10	Ph	126.9	129.2-130.1
H-5'	4.39	4.37	Ph	127.5	129.2-130.1
H-2'	3.94	4.79	Ph	131.9	129.2-130.1
H-1'	6.05	6.15	C-4'	78.6	81.4
CH3	1.96	1.34	C-3'	74.3	76.2
CH3	1.12	1.34	C-5'	71.8	74.4
CH3	1.00	1.34	C-2'	77.4	74.9
OCH_2-2'	3.97	4.25, 4.22	C-1'	72.2	81.6
OCH_2-2'	4.54	4.45, 4.42	C-6'	16.1	18.6
OCH_2-4'	4.29	4.59, 4.56, 4.56, 4.53	OCH_2-2'	70.6	72.7
OCH_2-4'	4.48	4.59, 4.56, 4.56, 4.53	OCH_2-4'	71.0	73.6
OCH_2-3'	4.50	4.75, 4.72, 4.70, 4.67	OCH_2-3'	74.6	74.1
OCH_2-3'	4.75	4.75, 4.72, 4.70, 4.67	Ph-Cq	136.8	139.7
Ph-2'	6.45	6.91	Ph	128.7	129.2-130.1
Ph-2'	7.22	7.40-7.27	Ph	127.8	129.2-130.1
Ph-2'	7.00	6.91	Ph	126.6	129.2-130.1
Ph-2'	7.20	7.12-7.05	Ph	126.4	129.2-130.1
Ph-2'	7.17	7.12-7.05	Ph	126.7	129.2-130.1
Ph-3'	7.53	7.40-7.27	Ph-Cq	137.6	139.8
Ph-3'	7.13	7.12-7.05	Ph	129.4	129.2-130.1
Ph-3'	7.43	7.40-7.27	Ph	129.0	129.2-130.1
Ph-3'	7.29	7.40-7.27	Ph	126.9	129.2-130.1
Ph-3'	7.36	7.40-7.27	Ph	126.7	129.2-130.1
Ph-4'	7.58	7.40-7.27	Ph	126.9	129.2-130.1

Ph-4'	7.45	7.40-7.27	Ph-Cq	137.4	138.8
Ph-4'	7.46	7.40-7.27	Ph	129.5	129.2-130.1
Ph-4'	7.43	7.40-7.27	Ph	129.1	129.2-130.1
Ph-4'	7.41	7.40-7.27	Ph	127.0	129.2-130.1
			Ph	126.8	129.2-130.1
			Ph	126.9	129.2-130.1

Table S5. NMR chemical shifts calculated for compound **12**, "conformer a".

H (paper)	^{1}H δ calc	^{1}H δ exp	C (paper)	^{13}C δ calc	^{13}C δ exp
H-2	8.47	8.75	C-4	148.5	153.5
H-8	7.87	8.59	C-5	119.0	125.0
NH	8.67	7.57-7.54	C-6	147.6	151.3
ortho PhCO	7.83	8.07	C-2	150.1	153.5
ortho PhCO	8.02	8.07	C-8	138.4	144.7
meta PhCO	7.52	7.57-7.54	COPh	162.7	168.3
meta PhCO	7.54	7.57-7.54	Cq, Ph	133.6	134.0
para PhCO	7.63	7.65	Ph	124.8	120.2
H-1'	6.22	6.30	Ph	128.2	129.8
H-2'	5.27	6.26	Ph	126.9	123.3
H-3'	5.38	5.65	Ph	127.5	129.5
H-4'	4.62	5.01	Ph	131.9	134.0
H-5'	4.13	4.28	C-3'	66.7	70.3
CH_3-6'	1.36	1.41-2.14	C-2'	67.8	68.9
CH_3-6'	1.34	1.41-2.14	C-1'	70.4	80.3
CH_3-6'	2.37	1.41-2.14	C-5'	74.4	73.1
CH_3	1.59	1.41-2.14	C-4'	71.2	73.2
CH_3	1.52	1.41-2.14	C-6'	15.3	17.2
CH_3	1.88	1.41-2.14	CO	169.0	171.2
CH_3	1.91	1.41-2.14	CH_3	20.2	20.4
CH_3	2.36	1.41-2.14	CO	169.5	171.2
CH_3	2.43	1.41-2.14	CH_3	20.8	20.6

CH_3	2.35	1.41-2.14	CO	169.0	171.2
CH_3	2.35	1.41-2.14	CH_3	20.9	20.7
CH_3	1.89	1.41-2.14			

Table S6. NMR chemical shifts calculated for compound **12**, “conformer b”.

H (paper)	^{1}H δ calc	^{1}H δ exp	C (paper)	^{13}C δ calc	^{13}C δ exp
H-2	8.44	8.75	C-4	150.7	153.5
H-8	7.92	8.59	C-5	121.8	125.0
NH	7.72	7.57-7.54	C-6	148.0	151.3
ortho PhCO	7.88	8.07	C-2	149.5	153.5
ortho PhCO	8.03	8.07	C-8	138.5	144.7
meta PhCO	7.51	7.57-7.54	COPh	164.0	168.3
meta PhCO	7.53	7.57-7.54	Cq, Ph	132.4	134.0
para PhCO	7.64	7.65	Ph	126.1	120.2
H-1'	6.29	6.30	Ph	128.1	129.8
H-2'	5.32	6.26	Ph	126.9	123.3
H-3'	5.39	5.65	Ph	127.2	129.5
H-4'	4.63	5.01	Ph	132.2	134.0
H-5'	4.12	4.28	C-3'	66.8	70.3
CH_3-6'	1.36	1.41-2.14	C-2'	67.4	68.9
CH_3-6'	1.36	1.41-2.14	C-1'	70.2	80.3
CH_3-6'	2.38	1.41-2.14	C-5'	74.4	73.1
CH_3	1.59	1.41-2.14	C-4'	71.2	73.2
CH_3	1.54	1.41-2.14	C-6'	15.4	17.2
CH_3	1.89	1.41-2.14	CO	169.0	171.2
CH_3	1.91	1.41-2.14	CH_3	20.3	20.4
CH_3	2.36	1.41-2.14	CO	169.5	171.2
CH_3	2.43	1.41-2.14	CH_3	20.8	20.6
CH_3	2.35	1.41-2.14	CO	169.0	171.2
CH_3	2.35	1.41-2.14	CH_3	20.9	20.7
CH_3	1.88	1.41-2.14			

Table S7. NMR chemical shifts calculated for compound **15**.

H (paper)	^{1}H δ calc	^{1}H δ exp	C (paper)	^{13}C δ calc	^{13}C δ exp
H-2	8.36	8.59	C-4	148.8	153.0
H-8	7.87	8.41	C-5	119.1	125.1
NH	8.77	7.41-7.24	C-6	147.4	151.0
ortho PhCO	7.91	8.09	C-2	149.7	153.0
ortho PhCO	8.05	8.09	C-8	139.1	145.1
meta PhCO	7.54	7.54	CONH	162.5	168.1
meta PhCO	7.55	7.54	Ph-Cq	133.6	138.5
para PhCO	7.64	7.63	Ph	124.7	138.5-128.7
H-1'	6.07	6.17	Ph	128.2	138.5-128.7
H-2'	4.13	4.82	Ph	126.9	138.5-128.7
H-3'	4.66	4.10	Ph	127.5	138.5-128.7
H-4'	3.65	3.64	Ph	131.9	138.5-128.7
H-5'	4.06	4.37	C-3'	70.2	76.0
CH_3	1.03	1.35	C-2'	77.8	74.6
CH_3	1.02	1.35	C-1'	72.4	81.3
CH_3	1.98	1.35	C-5'	77.0	74.1
CH_2Ph-2'	4.18	4.26, 4.23	C-4'	78.7	81.1
CH_2Ph-2'	4.75	4.45, 4.42	C-6'	16.6	18.3
Ph-2'	7.30	7.27-7.24, 7.15-7.04, 6.93	CH_2Ph	70.9	72.4
Ph-2'	6.46	7.27-7.24, 7.15-7.04, 6.93	Ph-Cq	137.0	139.4
Ph-2'	7.24	7.27-7.24, 7.15-7.04, 6.93	Ph	127.8	138.5-128.7
Ph-2'	7.02	7.27-7.24, 7.15-7.04, 6.93	Ph	128.6	138.5-128.7
Ph-2'	7.20	7.27-7.24, 7.15-7.04, 6.93	Ph	126.4	138.5-128.7

CH_2Ph-3'	4.83	4.75, 4.72	Ph	126.7	138.5-128.7
CH_2Ph-3'	4.54	4.70, 4.67	Ph	126.7	138.5-128.7
Ph-3'	7.16	7.27-7.24, 7.15-7.04, 6.93	CH_2Ph	74.3	73.8
Ph-3'	7.53	7.27-7.24, 7.15-7.04, 6.93	Ph-Cq	137.6	153.4
Ph-3'	7.30	7.27-7.24, 7.15-7.04, 6.93	Ph	129.0	138.5-128.7
Ph-3'	7.43	7.27-7.24, 7.15-7.04, 6.93	Ph	129.4	138.5-128.7
Ph-3'	7.36	7.27-7.24, 7.15-7.04, 6.93	Ph	126.7	138.5-128.7
CH_2Ph-4'	4.42	4.56, 4.53	Ph	126.9	138.5-128.7
CH_2Ph-4'	4.73	4.69, 4.57	Ph	126.9	138.5-128.7
Ph-4'	7.53	7.27-7.24, 7.15-7.04, 6.93	CH_2Ph	71.7	73.3
Ph-4'	7.53	7.27-7.24, 7.15-7.04, 6.93	Ph-Cq	137.4	139.5
Ph-4'	7.45	7.27-7.24, 7.15-7.04, 6.93	Ph	129.6	138.5-128.7
Ph-4'	7.44	7.27-7.24, 7.15-7.04, 6.93	Ph	128.9	138.5-128.7
Ph-4'	7.41	7.27-7.24, 7.15-7.04, 6.93	Ph	127.0	138.5-128.7
			Ph	126.8	138.5-128.7
			Ph	126.9	138.5-128.7

Molecular Electrostatic Potential Maps

Compound **5**

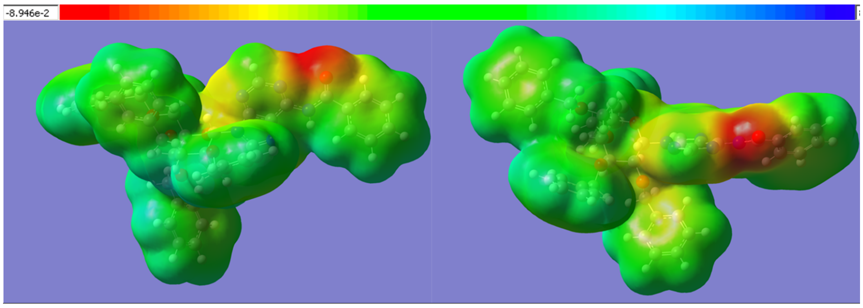

Compound **6**

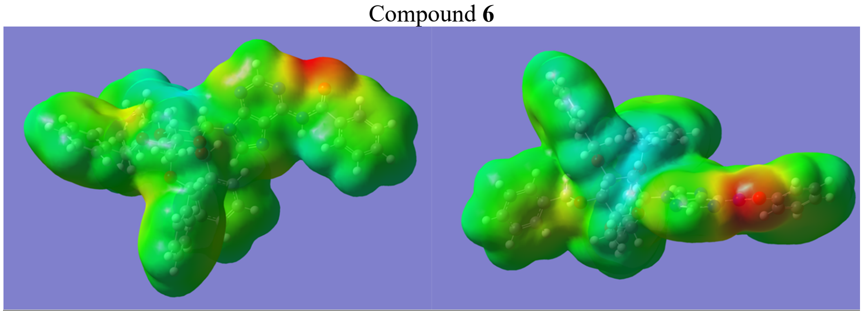

Compound **7**

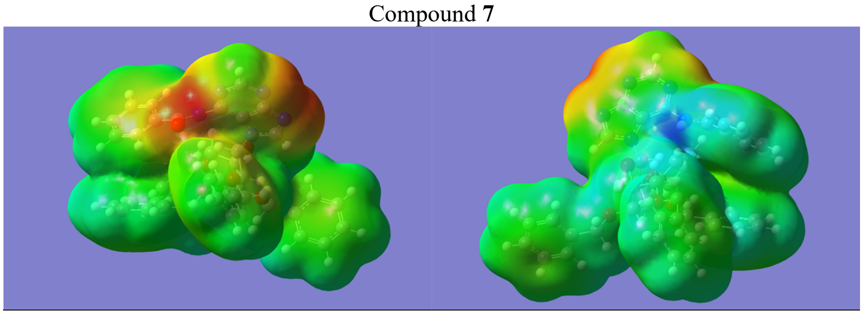

Compound **9**

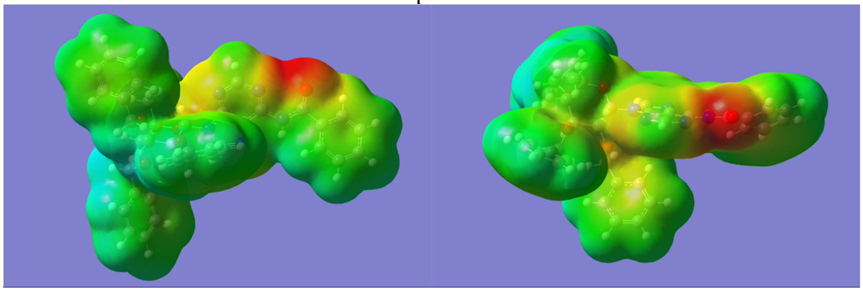

Figure S48. Different views of the electrostatic potential maps for compounds **5**, **6**, **7** and **9.**

Compound **12a**

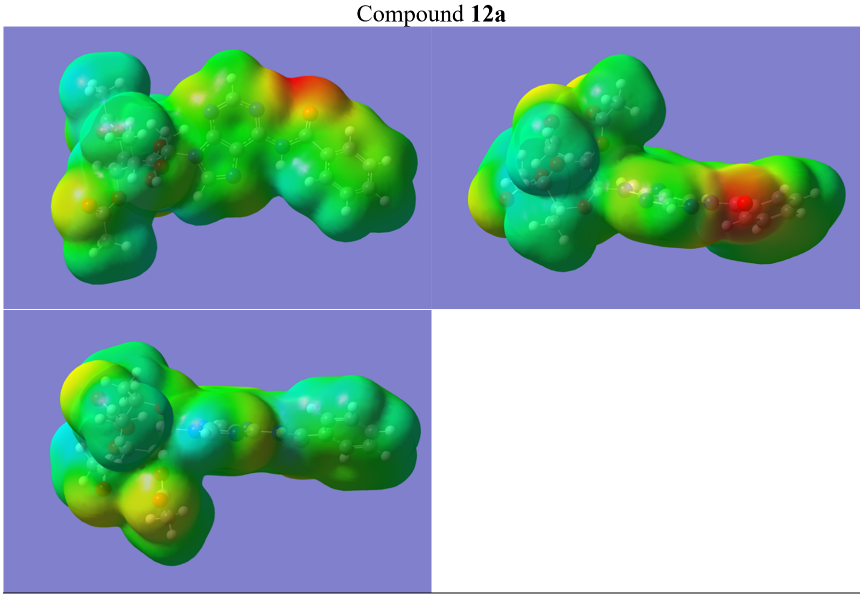

Compound **12b**

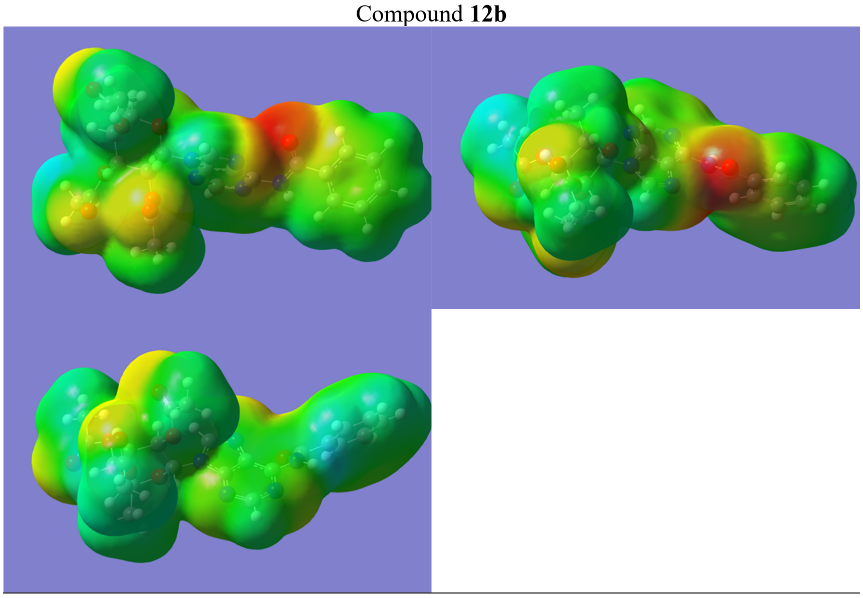

Figure S49. Different views of the electrostatic potential maps for conformers **12**a and **12**b.

Compound **15**

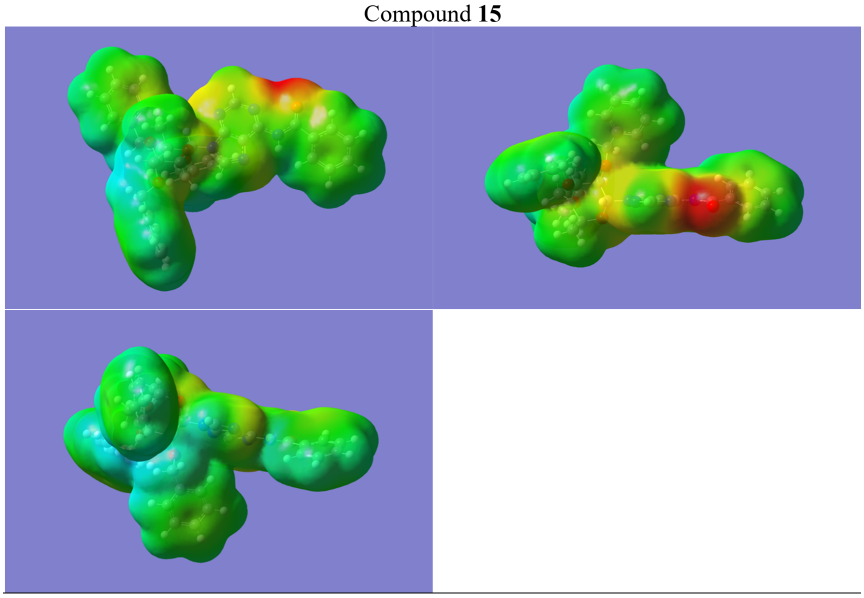

Figure S50. Different views of the electrostatic potential maps for compound **15**.

Energy differences between the most stable conformers found for compound 12 (α and β anomers)

Table S8. Energy differences between conformers "a" and "b" for compound 12 (α and β) and between compound 12a (α anomer) and 12b (β anomer).

Compound 12: α anomer			
conformer	E / kcal.mol^{-1}	ΔE(a-b) / kcal.mol^{-1}	Boltzmann population
a	-1132494.89	-0.55	0.72
b	-1132494.34		0.28
Compound 12: β anomer			
conformer	E / kcal.mol^{-1}	ΔE(a-b) / kcal.mol^{-1}	Boltzmann population
a	-1132488.59	-1.51	0.93
b	-1132487.08		0.07
Compound 12: α and β anomer			
Anomer	E / kcal.mol^{-1}	ΔE(α-β) / kcal.mol^{-1}	Boltzmann population
α	-1132494.89	-6.30	1.00
β	-1132488.59		0.00
